# Human milk variation is shaped by maternal genetics and impacts the infant gut microbiome

**DOI:** 10.1016/j.xgen.2024.100638

**Published:** 2024-09-11

**Authors:** Kelsey E. Johnson, Timothy Heisel, Mattea Allert, Annalee Fürst, Nikhila Yerabandi, Dan Knights, Katherine M. Jacobs, Eric F. Lock, Lars Bode, David A. Fields, Michael C. Rudolph, Cheryl A. Gale, Frank W. Albert, Ellen W. Demerath, Ran Blekhman

**Affiliations:** 1 Department of Genetics, Cell Biology, and Development, University of Minnesota, Minneapolis, MN, USA; 2 Division of Neonatology, Department of Pediatrics, University of Minnesota Medical School, Minneapolis, MN, USA; 3 Department of Pediatrics, University of California, San Diego, La Jolla, CA, USA; 4 BioTechnology Institute, College of Biological Sciences, University of Minnesota, Minneapolis, MN, USA; 5 Department of Computer Science and Engineering, University of Minnesota, Minneapolis, MN, USA; 6 Department of Obstetrics, Gynecology and Women’s Health, Division of Maternal-Fetal Medicine, University of Minnesota Medical School, Minneapolis, MN, USA; 7 Division of Biostatistics & Health Data Science, University of Minnesota School of Public Health, Minneapolis, MN, USA; 8 Human Milk Institute (HMI) and Mother-Milk-Infant Center of Research Excellence (MOMI CORE), University of California, San Diego, La Jolla, CA, USA; 9 Department of Pediatrics, University of Oklahoma Health Sciences Center, Oklahoma City, OK, USA; 10 Harold Hamm Diabetes Center, Department of Physiology, the University of Oklahoma Health Sciences Center, Oklahoma City, OK, USA; 11 Division of Epidemiology and Community Health, University of Minnesota School of Public Health, Minneapolis, MN, USA; 12 Section of Genetic Medicine, Division of Biological Sciences, University of Chicago, Chicago, IL, USA

**Keywords:** human milk, breastfeeding, lactation, eQTL, microbiome, nutrition

## Abstract

Human milk is a complex mix of nutritional and bioactive components that provide complete nourishment for the infant. However, we lack a systematic knowledge of the factors shaping milk composition and how milk variation influences infant health. Here, we characterize relationships between maternal genetics, milk gene expression, milk composition, and the infant fecal microbiome in up to 310 exclusively breastfeeding mother-infant pairs. We identified 482 genetic loci associated with milk gene expression unique to the lactating mammary gland and link these loci to breast cancer risk and human milk oligosaccharide concentration. Integrative analyses uncovered connections between milk gene expression and infant gut microbiome, including an association between the expression of inflammation-related genes with milk interleukin-6 (IL-6) concentration and the abundance of *Bifidobacterium* and *Escherichia* in the infant gut. Our results show how an improved understanding of the genetics and genomics of human milk connects lactation biology with maternal and infant health.

## Highlights

• Human milk transcriptomes reveal factors shaping the lactating mammary gland
• eQTL analysis identified tissue-shared and milk-specific genetic influences
• Milk eQTLs associated with milk composition and maternal breast cancer risk
• Milk with a signature of inflammation correlated with the infant gut microbiome

## Graphical abstract

**Figure:**
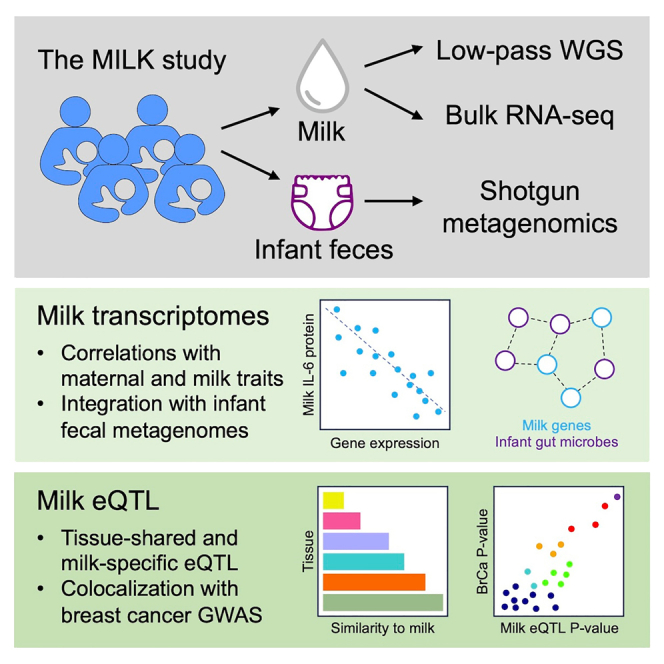

## Teaser

Human milk has positive health impacts for lactating parents and infants, and milk composition varies across individuals. Johnson et al. perform an eQTL study of human milk cells and link the milk transcriptome to maternal traits, milk composition, and the infant gut microbiome.

## Introduction

Lactation is a defining trait of mammals and has been essential for our species throughout human evolution.^1^ Today, breastfeeding is recommended as the exclusive mode of feeding for infants, given its documented health benefits for both mothers and infants.^2^ The nutritional significance of human milk stems from hundreds of milk constituents, including macro- and micro-nutrients, immune factors, hormones, oligosaccharides, and microbes.^3^ Maternal factors such as diet, health status, and genetics shape variation in milk composition across lactating women^4^^,^^5^; however, the role of maternal genetics in shaping milk composition is particularly understudied. A small number of studies suggest important relationships between maternal genotype, milk composition, and infant health.^6^ For example, maternal secretor status, determined by the *FUT2* gene, is linked to human milk oligosaccharide (HMO) composition.^7^ HMOs are sugars in human milk that cannot be digested by the infant but promote the growth of beneficial microbes in the infant gut and may provide additional immunological and metabolic benefits.^8^ In addition to HMOs, variation in other milk components, such as fatty acids, has been linked to the infant gut microbiome,^9^^,^^10^ and breastfeeding (vs. formula feeding) is one of the strongest factors shaping the infant gut microbiome.^11^^,^^12^ The abundance of certain microbes in the infant gut, particularly *Bifidobacterium*, has been linked to health outcomes in infancy and later childhood.^13^ Thus, the composition of the infant gut microbiome represents a key outcome through which human milk promotes infant health. Here, we combine maternal clinical and milk composition data with maternal whole-genome sequences, milk transcriptomes, and infant fecal metagenomics to characterize genetic influences on gene regulation in milk and identify pathways linking milk gene expression with milk composition and infant gut health. The results advance our knowledge of the complex molecular and physiological relationships connecting mother, milk, and infant.^14^

## Results

### Milk gene expression correlates with maternal traits and milk composition in a healthy, successfully lactating cohort

**Figure 1:**
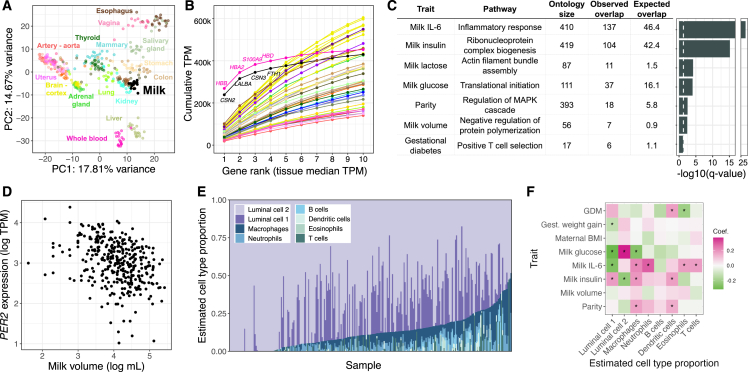
Overview of gene expression in human milk (A) Principal-component analysis of transcriptomes from a subset of GTEx tissues and milk. 19 random samples were chosen from each tissue. PCs were calculated using the 1,000 most variable genes within GTEx, and then milk samples were projected onto the GTEx samples. An equivalent plot including all GTEx tissues is shown in Figure S5. (B) Cumulative TPM (transcripts per million) of the top 10 genes by median TPM for milk and GTEx tissues. The color scheme is the same as in (A). (C) Gene Ontology enrichment of genes with expression correlated to maternal and milk traits. The most significant term for each trait is shown (STAR Methods). The dashed white vertical line denotes a q value of 0.05. (D) Correlation between milk volume (from standardized electric breast pump expression during a study visit; STAR Methods) and *PER2* gene expression in milk. (E) Cell type proportion estimates generated using Bisque^27^ for transcriptomes from this study with reference milk single-cell RNA-seq from Nyquist et al.^17^ (F) Heatmap of regression coefficients between estimated cell type proportions (x axis) and maternal or milk traits (y axis) from a linear model including technical covariates (STAR Methods). ∗q < 10%. See also Figure S1 and Tables S2, S3, S5, and S7.

Human milk contains mammary epithelial luminal cells and a variety of immune cell types, including macrophages, lymphocytes, and granulocytes.^15^^,^^16^^,^^17^^,^^18^^,^^19^ A milk sample provides rich information on immune phenotypes and the biology of milk production, as RNA extracted from milk profiles the milk-producing cells in the lactating mammary gland.^15^^,^^16^^,^^20^^,^^21^ To characterize population-level variation in human milk gene expression, we performed bulk RNA sequencing on cell pellets from 1-month postpartum milk samples from 316 women in the Mothers and Infants Linked for Healthy Growth (MILK) study^22^^,^^23^^,^^24^ (Figures S1, S2, S3, and S4; Table S1). Comparison to gene expression data from human tissues obtained by the Genotype-Tissue Expression (GTEx) consortium^25^ showed that milk expression profiles clustered near other secretory tissues, such as pancreas, kidney, and colon (Figures 1A and S5). The three most highly expressed milk genes (*CSN2*, *LALBA*, and *CSN3*), which comprise a large proportion of milk transcripts,^15^ accounted for 34.5% of protein-coding transcripts in milk, reminiscent of the preponderance of hemoglobin transcripts typical in whole blood (Figure 1B).^25^ These three genes encode the major milk proteins beta- and kappa-casein (*CSN2* and *CSN3*) and lactalbumin (*LALBA*), an essential protein for lactose and HMO synthesis.^26^

To identify factors associated with the milk transcriptome, we tested for correlations between the expression of 12,006 genes in milk and 13 maternal or milk traits in *n* = 269 participant’s milk samples (or *n* = 171 for milk macronutrients; Tables S2, S3, and S4; Figures S6, S7, and S8). In this analysis, we used a gene-wise model testing for differences in each gene’s expression to maternal or milk traits and technical covariates (STAR Methods). Milk composition traits were measured from separate aliquots of the same milk samples as used for RNA sequencing (RNA-seq) (STAR Methods). Among maternal traits, gestational diabetes status and parity were correlated with expression of the most genes (gestational diabetes: 784 genes, parity: 172 genes at q < 10%; negative binomial generalized log-linear test; STAR Methods). Genes for which expression correlated with parity were enriched for pathways related to cell communication and the mitogen-activated protein kinase cascade, potentially reflecting persistent differences in mammary gland epigenetic states and remodeling during lactation in participants who had lactated previously^28^^,^^29^ (Figure 1C). Pre-pregnancy BMI and gestational weight gain, traits associated with delayed lactogenesis and breastfeeding challenges,^30^ were correlated with milk expression of just a few genes (<30 genes; Table S3). This weak relationship could be due to our study’s inclusion of only women who successfully breastfed for at least 1 month postpartum, thus excluding participants with difficulties initiating breastfeeding related to metabolic health. Milk concentrations of IL-6, glucose, insulin, and lactose and the total single breast milk expression volume produced at the study visit were each correlated with expression of hundreds of genes (q < 10%; Table S3). These milk trait-correlated genes were enriched for processes such as translation (milk insulin) and cytoskeleton organization (milk volume) (Figure 1C; Table S5). There was no significant interaction with maternal obesity status for any gene/trait pair after multiple test correction (STAR Methods; Table S6).

The gene for which expression was most significantly associated with expressed milk volume was the core circadian clock gene *PER2*. Higher *PER2* expression correlated with lower milk volume (log_2_ fold change = −0.22, q = 9.5 × 10^−9^; Figure 1D; Table S3). The relationship between *PER2* expression and milk volume was not driven by the time of day of milk expression (F test, *p* = 0.06; Figure S9; STAR Methods). It is notable that we observed this correlation even though milk volume is variable within individuals^31^ and was assessed in a single visit (STAR Methods). In addition to *PER2*, the circadian gene *RORC* was also associated with milk volume (log_2_ fold change = −0.10, q = 0.03). *PER2* plays a role in cell fate and ductal branching in the mammary gland in addition to its circadian function.^32^ Our observation suggests that differential expression of circadian clock genes in the mammary gland affects milk production in humans, possibly via regulation of milk production genes or by anatomical changes in the breast during lactogenesis.

Of all milk traits tested, glucose concentration was correlated with expression of the largest number of genes (1,634 genes at q < 10%; Table S3), followed by IL-6 protein and insulin concentrations (1,235 and 1,144 genes at q < 10%, respectively). Genes correlated with insulin and glucose concentrations were both strongly enriched for ribosomal proteins. Genes correlated with milk IL-6 concentration were enriched for immune pathways, with “inflammatory response” the most significantly enriched pathway (q = 4.1 × 10^−27^, Fisher’s exact test; Figure 1C), consistent with IL-6’s role as a marker of inflammation in the mammary gland.^33^ To estimate the contributions of different cell types to our milk bulk transcriptomes, we performed cell-type deconvolution using a milk single-cell RNA-seq reference panel (Figure 1E; STAR Methods).^17^^,^^27^ Consistent with previous studies, mammary epithelial cells were estimated to make up the majority of cells.^17^^,^^18^^,^^19^^,^^34^ The estimated proportion of several immune cell types were increased in milk samples with higher IL-6 concentration (e.g., neutrophils: multiple regression coefficient = 0.29, q = 3.4 × 10^−4^; macrophages: multiple regression coefficient = 0.22, q = 6.2 × 10^−3^; Figure 1F; Table S7), suggesting that the relationship between IL-6 concentration and immune gene expression is linked to a greater proportion of immune cells in milk.

### Genetic influences on gene expression in human milk

**Figure 2:**
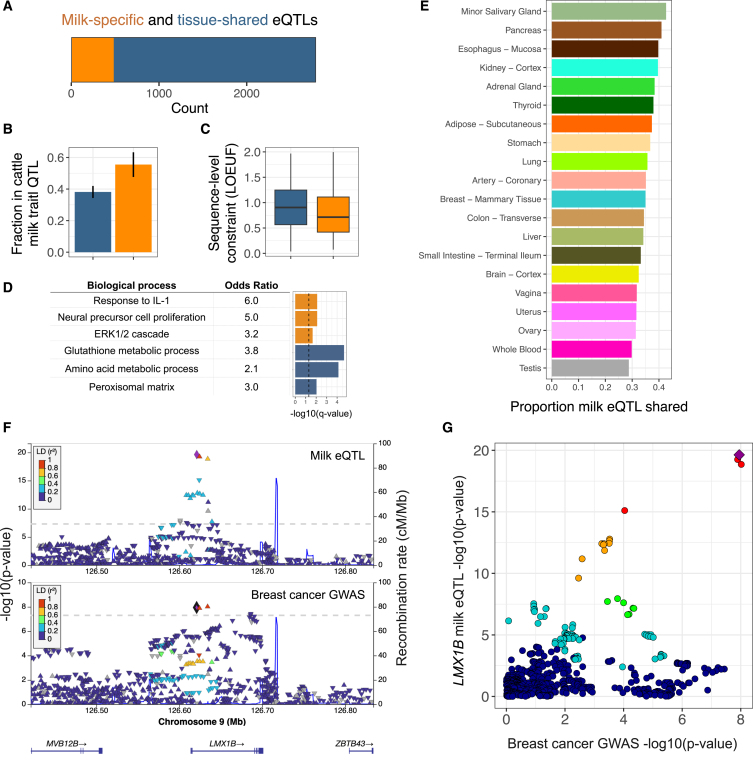
Genetic influences on gene expression in human milk (A) Counts of genes with milk-specific eQTLs (orange, genes with an eQTL signal that did not colocalize with any GTEx tissue; STAR Methods) vs. tissue-shared eQTLs (blue, genes with all milk eQTL signals colocalized with at least one GTEx tissue). (B) Fraction of genes in each category that overlapped with a milk trait QTL in the dairy cattle genome. Error bars represent a 95% confidence interval. (C) Distributions of sequence-level constraint, measured by the loss-of-function observed/expected upper bound fraction statistic.^38^ (D) Enriched Gene Ontologies for genes with milk-specific (orange) or tissue-shared (blue) eQTLs. The dashed vertical line denotes a q value of 5%. (E) Fraction of shared milk eQTLs with a subset of GTEx tissues, estimated with *mash*.^40^ (F) LocusZoom genetic associations in the *LMX1B* region with milk gene expression (top) and breast cancer risk (bottom). Each data point represents a SNP, plotted by its chromosomal location (x axis) and significance of association (y axis), with colors corresponding to linkage disequilibrium (r^2^) to the lead SNP for the milk eQTL, shown as a purple diamond. (G) Each point is a variant, plotted by the strength of association with milk gene expression (y axis) and breast cancer risk (x axis). Colors are the same as in (F), top, with a purple diamond representing the lead milk eQTL SNP. The pattern of variants in the top right suggests a shared underlying causal variant. See also Figures S13, S14, S15, S16, S17, S18, S19, and S20 and Tables S8, S9, S10, S11, S12, and S13.

Associations between genetic variation and gene expression can illuminate the molecular mechanisms underlying genetic influences on human traits,^35^ but this approach has not been applied to human milk. To identify associations between maternal genetic variation and milk gene expression, we generated low-pass whole-genome sequencing data and performed an expression quantitative trait locus (eQTL) scan in 230 unrelated human milk samples (STAR Methods). We identified a local eQTL (q < 5%) at 2,790 genes of 17,302 tested (Table S8; Figures S10, S11, and S12), with 45 genes showing evidence of multiple independent signals in conditional analysis (Table S9). Comparing milk eQTLs to those identified in 45 human tissues in the GTEx project,^25^ we partitioned our eQTLs as milk specific (*n* = 482) or shared with at least one other tissue (*n* = 2,308) by detecting milk-specific eQTL effects via statistical colocalization^36^^,^^37^ (Figure 2A; Table S10; STAR Methods). Genes with milk-specific eQTLs highlighted key biological pathways in the lactating mammary gland: production of caseins (e.g., the abundant milk proteins *CSN3* and *CSN1S1*), lactose synthesis (*LALBA*), lipogenesis (e.g., *ACSL1*, *LPL*, *IDH1*, and *LPIN1*), hormonal regulation (*INSR*), and immunity (e.g., *LYZ*, *MUC7*, and *CD68*) (Table S10). In addition, genes with milk-specific eQTLs were twice as likely as genes with eQTLs shared across multiple tissues to overlap genetic associations for milk traits in dairy cattle (odds ratio = 2.0, *p* = 1.7 × 10^−4^, two-sided Fisher’s exact test; Figure 2B; Table S11), a species for which there is far more known about genetic influences on lactation than in humans. This enrichment suggests that genes with milk-specific eQTLs are specifically important for milk biology. Genes with milk-specific eQTLs also tended to have more sequence-level constraint^38^ than tissue-shared eQTLs (*p* = 2.4 × 10^−6^, Wilcoxon rank-sum test; Figure 2C) and were enriched for pathways such as “regulation of ERK1 and ERK2 cascade” (Figure 2D; STAR Methods), which has a key role in mammary morphogenesis.^39^

To identify tissues for which genetic regulation of gene expression is most similar to milk, we estimated the proportion of shared eQTLs between milk and each GTEx tissue using *mash*^40^ (STAR Methods; Table S12). Milk shared the largest proportion of eQTLs with secretory tissues (e.g., minor salivary gland, pancreas, and esophagus), with a higher proportion shared than that observed for non-lactating breast tissue (Figures 2E and S13). These comparisons highlight the shared regulation of gene expression across secretory tissues and underscore the insufficiency of non-lactating breast tissue for studying gene expression programs necessary for lactation.

Epidemiological studies describe a complex relationship between lactation and breast cancer risk, with decreased or increased risk depending on age at first pregnancy and decreased lifetime risk associated with longer duration of lactation.^41^^,^^42^ Because the genetics of gene expression in the lactating mammary gland is distinct from that of non-lactating breast (Figure 2E), milk eQTLs provide unique functional annotations to genetic associations with breast cancer. Using colocalization analyses between all milk eQTLs and breast cancer genome-wide association study (GWAS) loci,^43^ we identified 7 loci with strong evidence of a shared causal variant (posterior probability of shared causal variant >0.9; Table S13; Figures S14, S15, S16, S17, S18, and S19). Of these milk eQTL-GWAS colocalizations, 4 had been nominated previously as a causal gene for breast cancer,^44^^,^^45^^,^^46^ and 2 were eQTLs for pseudogenes (Table S13). We identified a novel candidate gene at a breast cancer GWAS locus where a milk eQTL that increased expression of *LMX1B* was associated with increased cancer risk (Figures 2F and 2G). *LMX1B* does have not have a significant GTEx eQTL in mammary tissue.^25^ The milk *LMX1B* eQTL colocalized with one GTEx tissue at an eQTL for the tibial nerve (Figure S20). *LMX1B* is a transcription factor essential for normal development of limbs, kidneys, and ears.^47^

### Milk gene expression correlates with concentrations of HMOs

**Figure 3:**
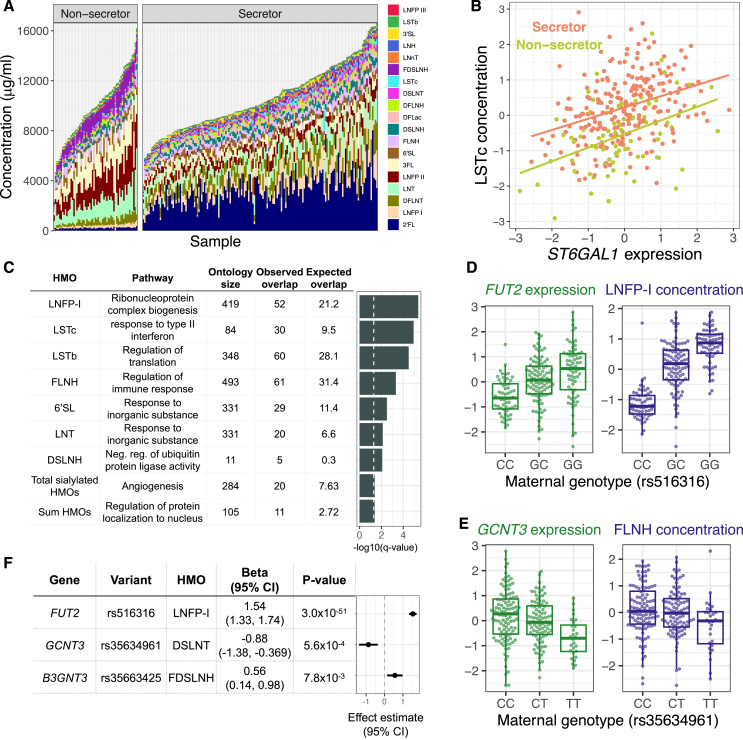
Effects of milk gene expression on HMO composition (A) HMO concentration (y axis) profiles for milk samples in our study (x axis), grouped by secretor status. (B) Correlation between *ST6GAL1* gene expression in milk and normalized LSTc concentration, colored by secretor status (log_2_ fold change = 0.32, *p* = 6.6 × 10^−8^, q = 1.5 × 10^−4^). (C) Gene Ontology enrichment of genes with expression correlated to a single HMO or HMO category. The most significant term for each HMO is plotted. The dashed vertical line denotes a q value of 5%. (D) Relationships between genotype at the lead SNP at the *FUT2* eQTL and *FUT2* expression in milk (green) or LNFP-I concentration (purple). LNFP-I concentrations are residuals after correcting for genetic PCs (STAR Methods). (E) Relationships between genotype at the lead SNP at the *GCNT3* eQTL and *GCNT3* expression in milk (green) or FLNH concentration (purple). FLNH concentrations are residuals after correcting for secretor status and genetic PCs (STAR Methods). (F) Estimates of the effect of milk gene expression of candidate HMO biosynthesis pathway genes on the abundance of HMOs from a Wald ratio test. Some genes had significant effects on more than one HMO (Table S18). The most significant HMO for each gene is plotted here. See also Figures S21, S22, and S23 and Tables S14, S15, S16, S17, and S18.

Maternal genetics play a strong role in shaping the concentration of HMOs,^7^ sugars in milk that are not digested by the infant but promote the growth of beneficial microbes in the infant gut. HMOs are synthesized in the mammary gland by addition of monosaccharides to a lactose molecule, but the glycosyltransferases catalyzing these reactions are largely uncharacterized.^48^ Secretor status, determined by the absence of a common nonsense variant in the fucosyltransferase 2 (*FUT2*) gene, strongly predicts the concentration of certain HMOs, with the presence of some HMOs entirely determined by secretor status.^7^ Utilizing 310 participants with both milk gene expression and 1-month HMO composition data, we observed distinct HMO profiles between secretors and non-secretors (Figures 3A and S21; see Table S14 for HMO definitions). We hypothesized that, beyond the strong effects of the secretor polymorphism, the expression of *FUT2* in milk would correlate with HMO concentrations within secretor individuals, reflecting variation in milk among women with a functional FUT2 enzyme. We observed nominally significant associations between *FUT2* expression and the concentration of three HMOs: 2′-fucosyllactose (beta = 0.12, *p* = 0.01; Figure S22), lacto-N-fucopentaose (LNFP)-II (beta = −0.12, *p* = 0.03; Figure S22), and lacto-N-hexaose (beta = 0.14, *p* = 0.04; Figure S22). This suggested that milk gene expression data could be useful for identifying critical genes for HMO biosynthesis. We tested for pairwise correlations between gene expression and 19 individual HMOs and the sums of all HMO concentrations, sialylated HMOs, and fucosylated HMOs while controlling for secretor status (STAR Methods). These 22 HMO traits were significantly correlated with expression of between 8 and 1,262 genes (q < 10%; Table S15), including known HMO biosynthesis genes, such as the sialyltransferase *ST6GAL1*,^48^ with the HMO sialyl-lacto-N-tetraose c (LSTc) (beta = 0.80, *p* = 6.6 × 10^−8^, q = 1.5 × 10^−4^; Figure 3B). The genes correlated with 6 of the HMO traits were enriched for pathways related to ribosomes, such as “cytosolic ribosome” enriched in genes correlated with the sum of all HMOs (Figure 3C; Table S16). Genes correlated with the HMO 6′-sialyllactose or the sum of sialylated HMOs were enriched for inflammation-related pathways such as “cytokine activity” (Table S16), consistent with previous evidence that sialylated HMOs were more abundant in women with mastitis compared to healthy women.^49^

HMO biosynthesis represents an ideal system to understand the effects of maternal genetics on milk composition via changes in gene expression, as gene expression from the relevant cell type (mammary epithelial cells) and HMO concentrations can be measured non-invasively in the same milk samples. Among 54 candidate glycosyltransferase genes,^48^ seven genes had significant milk eQTLs in our data (q < 5%; Table S17), which we used to test for associations between maternal genotypes at milk eQTL tag SNPs and HMO concentrations in 224 individuals with both data types. For three genes, we observed an association between genotype and between 1 and 13 HMOs (Table S18; q < 10%). These included the known association of *FUT2* with 13 HMOs (e.g., LNFP-I; Figure 3D) and an association between *GCNT3* and fucosyllacto-N-hexaose (FLNH) (Figure 3E). *GCTN3* was also linked to FLNH in our above analysis of correlations between gene expression and HMO concentrations (Table S15; Figure S23). *GCTN3* has been identified previously as the best candidate gene responsible for the addition of a β-1,6-linked N-acetylglucosamine to the lactose core, a step required for the biosynthesis of FLNH.^48^ For each eQTL-HMO pair (q < 10%), we then estimated the causal effect of modified gene expression on HMO concentration using a Wald ratio test (Figure 3F; Table S18). These results provide evidence of direct or indirect roles of specific glycosyltransferases in HMO biosynthesis in the lactating mammary gland.

### Milk gene expression is associated with the infant gut microbiome

**Figure 4:**
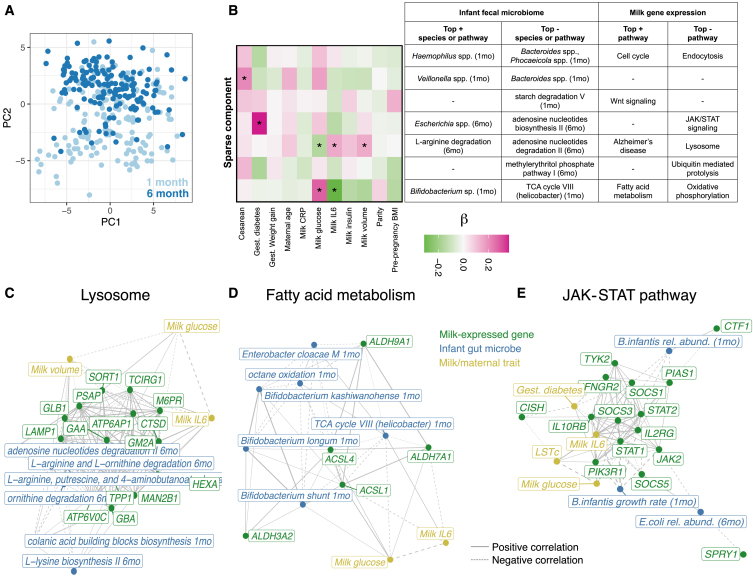
Interactions between milk gene expression and the infant fecal microbiome (A) Principal-component analysis of infant fecal microbiome metagenomic data, summarized at the taxonomic level, with each point representing a fecal sample and colors representing infant age (light blue, 1 month; dark blue, 6 months). (B) Sparse CCA integrating milk host gene expression and infant fecal microbial species or microbial genetic pathway relative abundance (at 1 or 6 months of age) identified seven significant sparse components (in rows). The heatmap on the left shows Spearman correlation coefficients between each mother/infant pair score for a given sparse component (rows) and maternal or milk traits (columns). The table lists the most highly weighted microbial taxon or genetic pathway and the most significantly enriched host gene set in milk gene expression. (+) or (−) indicates whether these features were positively or negatively weighted in the sparse component. (C and D) Network diagrams generated using the correlation matrix of infant fecal microbial species/pathways and milk-expressed host genes within an enriched pathway for two of the sparse components in (B). Line size corresponds to the absolute value of the correlation coefficient, and line type corresponds to negative (dashed) or positive (solid) correlations. Node color signifies milk-expressed host genes (green), infant fecal microbial pathways/taxa (green), or maternal/milk traits (yellow). Plotted edges had correlation *p* < 0.05. (E) Network diagram displaying correlations between milk IL-6 concentration, LSTc (HMO) concentration, JAK-STAT pathway genes expressed in milk, and *B. infantis* relative abundance and estimated growth rate in the infant gut at 1 month and *Escherichia coli* relative abundance at 6 months. JAK-STAT pathway genes were selected that had a significant correlation with *B. infantis* or *E. coli* abundance after multiple test correction (q < 10%). See also Figure S24 and Tables S19, S20, and S21.

Studies have found correlations between milk composition and variation in the infant gut microbiome.^9^^,^^10^^,^^50^^,^^51^ However, it is unclear how these correlations are shaped by maternal genetics and milk gene regulation. We hypothesized that, given milk gene expression reflects milk composition, it could be correlated with the infant gut microbiome. We profiled the fecal microbiome of infants in our study with metagenomic sequencing at 1 and 6 months postpartum (*n* = 146; Figures 4A and S24) and identified nine correlated sets of genes expressed in milk and microbial taxa or pathways present in the infant gut at 1 or 6 months postpartum using sparse canonical correlation analysis (CCA)^52^^,^^53^ (STAR Methods; Figure 4B; Table S19). Using pathway enrichment analysis, we identified relevant biological processes in these milk-expressed gene sets correlated with the infant fecal microbiome (Table S20). For example, milk expression of lysosome genes was negatively correlated with the abundance of microbial genetic pathways related to amino acid degradation in the infant gut at 6 months (Figure 4C), and expression of fatty acid metabolism genes in milk was positively correlated with the abundance of species of *Bifidobacterium* in the infant gut at 1 month (Figure 4D). Lysosomes are involved in mammary gland remodeling and involution,^54^^,^^55^ and human milk fats can act as prebiotics to support growth of commensal bacteria in the infant gut, including *Bifidobacterium*.^56^ These links between milk gene expression and the infant gut microbiome nominate biological pathways through which normal variation in human milk composition may influence the infant gut microbiome.

The sparse CCA algorithm identified species of *Escherichia* at 6 months in the infant gut as negatively correlated with milk-expressed genes in the Janus kinase (JAK)-signal transducer and activator of transcription (STAT) pathway, which is a key regulator of both milk production and mammary inflammation.^57^ This sparse component was also correlated with gestational diabetes status (Figure 4B). We noted that the component highlighting abundance of *Bifidobacterium* in infants at 1 month was also enriched for milk-expressed genes in inflammation-related pathways (Table S20) and correlated with milk concentrations of IL-6 and glucose. *Bifidobacterium* spp. are abundant microbes in the breastfed infant gut that promote beneficial health outcomes, particularly *B. infantis*.^58^^,^^59^ *Escherichia* spp. are abundant in the infant gut, at higher levels in full term (vs. preterm) infants, and increase in abundance after the introduction of solid foods.^60^ Given our observation that genes in the JAK-STAT pathway were significantly correlated with milk IL-6 concentration (Table S3), we further examined the relationships between milk expression of JAK-STAT pathway genes, gestational diabetes, milk composition, and infant fecal *Escherichia* and *Bifidobacterium* levels. Given the well-known relevance of *B. infantis* to infant health, we also computationally inferred *B. infantis* growth rates in samples from 1-month-old infants (STAR Methods), an additional aspect of microbial community dynamics that varies across individuals and is relevant to disease.^61^ Both infant fecal *B. infantis* growth rate and relative abundance were negatively correlated with milk expression of JAK-STAT pathway genes, most significantly *SOCS3* (growth rate: Pearson’s r = −0.52, *p* = 1.4 × 10^−4^; relative abundance: r = −0.19, *p* = 0.02; Figure 4E; Table S21). *SOCS3* encodes a key element of the mammary anti-inflammatory response to bacterial mastitis^62^ and is most highly expressed in the immune cells in milk.^17^ Thus, the correlation between increased JAK-STAT signaling in milk and lower *B. infantis* abundance and growth in the infant gut could be related to an immune response to infection of the mammary gland.

## Discussion

Here, we generated and integrated multiple omics datasets within a cohort of exclusively breastfeeding mother-infant pairs, leveraging the milk transcriptome as a readout of the biology of milk production. Our results highlight how an improved understanding of the genetics and genomics of human milk reveals connections with maternal and infant health.

A consistent theme across our results was a link between mammary inflammation-related gene expression, milk composition, and the infant gut microbiome. Milk IL-6 concentration was correlated with milk gene expression across hundreds of genes (Table S3). Genes correlated with the concentration of multiple HMOs in milk were enriched for inflammation-related pathways (Figure 3C; Table S16), and expression of inflammation-related genes in milk was inversely correlated with the abundance and growth of *Bifidobacterium* in the infant gut at 1 month and *Escherichia* at 6 months (Figure 4E). All participants in our study were exclusively breastfeeding and did not report symptoms of mastitis (infection of the mammary gland) at the time of milk collection. Subclinical mastitis is prevalent across human populations and is associated with differences in milk composition.^63^^,^^64^^,^^65^^,^^66^ Thus, our results suggest that mammary inflammation, even when unnoticeable to the lactating individual, is a primary driver of variation in milk composition with potential effects on the infant gut microbiome.

Combining milk gene expression with maternal genetic variation, we identified numerous novel milk-specific eQTLs, which can now be used as targets for investigation of the effects of gene expression on milk production and composition and infant and maternal health. For example, combining our milk eQTLs with breast cancer GWAS summary statistics, we provide the first functional evidence connecting *LMX1B* expression to a nearby breast cancer GWAS locus (Figures 2F and 2G). Functional evidence for this GWAS locus had previously been missing, as this gene does not have an eQTL in GTEx mammary tissue and, thus, may only be detectable during lactation. In an analysis of single-cell RNA-seq across human tissues, *LMX1B* was most highly expressed in salivary and breast glandular cells.^67^ In addition, hypomethylation at a CpG island in *LMX1B* in human milk samples was associated with subsequent diagnosis of breast cancer in an epigenome-wide association study,^68^ suggesting higher expression correlated with breast cancer risk, which is concordant with the direction of effect in our results.

The importance of breastfeeding, especially in underdeveloped countries, is widely acknowledged, but the long-term health effects in modern high-income contexts are less concrete.^2^ Similarly, the causal effects of differences in milk composition for breastfed infants are underexplored due to the ethical and logistical impediments to performing randomized trials of infant nutrition. The field of human genetics has been hugely successful in identifying genetic effects on molecular and complex traits and has leveraged these associations to improve our understanding of disease pathophysiology, identify drug candidates, and interrogate causal relationships impacting human health. However, traits related to women’s health generally have been overlooked by this area of research, and human milk and lactation are glaring examples of this neglect. Fortunately, milk represents an easily obtained non-invasive biospecimen, aiding our ability to close this gap. Our study provides a step toward leveraging modern human genomics techniques to characterize the factors that shape milk composition and understand how this composition impacts infant and maternal health.

### Limitations of the study

While our study introduced a framework for integrating multiple and diverse data types in the mother/milk/infant triad, it is limited by sample size, particularly of our milk composition phenotypes and infant fecal microbiome data. Additionally, the MILK study is predominantly composed of participants who self-identify as white and non-Hispanic (∼85%). Thus, our analysis was limited to genetic variants common in participants of European ancestry, and our eQTL results may not be generalizable to other ancestry groups. Last, we studied mature milk collected 1 month postpartum, which did not allow us to assess genetic effects on colostrum or milk produced at other points in lactation.

## Resource availability

### Lead contact

Further information and requests for resources should be directed to and will be fulfilled by the lead contact, Kelsey Johnson (kej@umn.edu).

### Materials availability

This study did not generate new unique reagents.

### Data and code availability

• RNA-seq quantifications, infant fecal metagenomic abundances, HMO concentrations, milk eQTL summary statistics, and study metadata are available at figshare and are publicly available as of the date of publication. DOIs are listed in the key resources table.
• Maternal genotypes and raw RNA and DNA sequencing data have been deposited at dbGaP and are available under controlled access in compliance with the study IRB. Use of the data is limited to health/medical/biomedical purposes, including methods development and excluding the study of population origins. Data access is provided by dbGaP (https://www.ncbi.nlm.nih.gov/gap/) for certified investigators and does not require local IRB approval. Accession numbers are listed in the key resources table.
• Raw infant fecal metagenomic sequencing data have been deposited at the Sequence Read Archive (https://www.ncbi.nlm.nih.gov/sra) and are publicly available as of the date of publication. Accession numbers are listed in the key resources table.
• This paper does not report original code.
• Any additional information required to reanalyze the data reported in this paper is available from the lead contact upon request.

## Acknowledgments

The authors would like to acknowledge and thank all the participants and health care providers who contributed to the MILK study and MILK study teams, particularly Dr. Elyse Kharbanda and Dr. Kristin Palmsten at HealthPartners Institute, Bloomington, MN, for their leadership in participant recruitment at the Minnesota site. We thank Katy Duncan, Laurie Foster, Tipper Gallagher, and all MILK study staff and participants for their contributions and members of the Albert and Blekhman labs for helpful discussions related to this project. This work was supported by the resources and staff at the 10.13039/100017638University of Minnesota Genomics Center (https://genomics.umn.edu). This work was carried out in part by resources provided by the Minnesota Supercomputing Institute (https://www.msi.umn.edu/) and the Clinical and Translational Research Services support team at the Clinical and Translational Science Institute at the University of Minnesota (supported by grant number UL1TR002494 from the National Institutes of Health's National Center for Advancing Translational Sciences). This study was supported by a 10.13039/100007249University of Minnesota Department of Pediatrics Masonic Cross-Departmental Research Grant (to F.W.A., R.B., E.W.D., and C.A.G.), University of Minnesota Masonic Children’s Hospital Research Fund Award (to C.A.G., E.W.D., and D.K.), 10.13039/100000002NIH/NICHD grant R01HD109830 (to R.B., E.W.D., and C.A.G.), NIH/NICHD grant R21HD099473 (to C.A.G.), NIH/10.13039/100000057NIGMS grant R35GM124676 (to F.W.A.), a Pew Biomedical Fellowship (to F.W.A.), and a University of Minnesota Office of Academic and Clinical Affairs Faculty Research Development Grant (to C.A.G., E.W.D., K.M.J., and D.K.). The MILK study, which provided the cohort and milk samples for this study, was supported by NIH/NICHD grant R01HD080444 (to E.W.D. and D.A.F.). K.E.J. was supported by NIH/NICHD F32HD105364 and NIH/10.13039/100000072NIDCR T90DE0227232.

## Author contributions

Conceptualization, K.E.J., F.W.A., E.W.D., and R.B.; formal analysis, K.E.J., T.H., and M.A.; funding acquisition, K.E.J., D.K., K.M.J., E.F.L., L.B., D.A.F., C.A.G., F.W.A., E.W.D., and R.B.; investigation, K.E.J., T.H., E.W.D., K.M.J., D.A.F., A.F., and N.Y.; supervision, K.E.J., L.B., M.C.R., C.A.G., F.W.A., E.W.D., and R.B.; writing – original draft, K.E.J.; writing – review and editing, K.E.J., T.H., E.F.L., L.B., M.C.R., C.A.G., F.W.A., E.W.D., and R.B.

## Declaration of interests

The authors declare no competing interests.

## STAR★Methods

### Key resources table

**Table:** 

REAGENT or RESOURCE	SOURCE	IDENTIFIER
**Software and algorithms**		

STAR v2.7.1a	Dobin et al.^69^	https://github.com/alexdobin/STAR
RNA-SeQC73 v2.3.4	DeLuca et al.^70^	https://github.com/francois-a/rnaseqc
R package: DESeq2 v1.30.0	Love et al.^71^	https://bioconductor.org/packages/release/bioc/html/DESeq2.html
BCFtools v1.6	Danecek et al.^72^	https://www.htslib.org/download/
PLINK v1.90b6.10	Purcell et al.^73^	https://www.cog-genomics.org/plink/
R package: edgeR v3.32.1	Robinson et al.^74^	https://bioconductor.org/packages/release/bioc/html/edgeR.html
R package: topGO	Alexa et al.^75^	https://bioconductor.org/packages/release/bioc/html/topGO.html
BisqueRNA R package	Jew et al.^27^	https://github.com/cran/BisqueRNA
APEX toolkit	Quick et al.^76^	https://github.com/corbinq/apex
R package: coloc	Giambartolomei et al.^77^	https://cloud.r-project.org/web/packages/coloc/index.html
R package: mashR	Urbut et al.^40^	https://github.com/stephenslab/mashr
BURST version 0.99.7f96	Al-Ghalith et al.^78^	https://github.com/knights-lab/BURST
MetaPhlAn v3.0.7	Beghini et al.^79^	https://huttenhower.sph.harvard.edu/metaphlan/
Sparse canonical components analysis code	Priya et al.^53^	https://github.com/blekhmanlab/host_gene_microbiome_interactions
CoPTR	Joseph et al.^61^	https://github.com/tyjo/coptr

**Deposited data**		

Milk RNA-sequencing data	This paper	dbGaP: phs003408.v1.p1
Milk DNA-sequencing data and genotypes	This paper	dbGaP: phs003408.v1.p1
Infant fecal metagenomic sequencing data	This paper	SRA: PRJNA1019702
Milk transcriptome quantifications, infant fecal metagenome abundances, milk eQTL summary statistics, HMO concentrations, additional metadata	This paper	https://figshare.com/collections/Johnson_et_al_human_milk_multi-omics/7371256
GTEx RNA-sequencing quantifications and eQTL summary statistics	GTEx Portal^25^	https://gtexportal.org/home/downloads/adult-gtex/overview
1000 Genomes genotypes	Byrska-Bishop et al.^80^	https://www.internationalgenome.org/data-portal/data-collection/30x-grch38
Single-cell human milk RNA-seq data	Nyquist et al.^17^	https://singlecell.broadinstitute.org/single_cell/study/SCP1671/cellular-and-transcriptional-diversity-over-the-course-of-human-lactation
Breast cancer GWAS summary statistics	Zhang et al.^43^	http://bcac.ccge.medschl.cam.ac.uk/

### Experimental model and study participant details

#### Human study participants

This observational study comprised female adults recruited prenatally in the United States and their infants. Individual level demographic information and covariates are available in supplementary tables and on figshare (see key resources table). The Institutional Review Boards of the University of Oklahoma, the University of Minnesota, and the HealthPartners Institute approved this study (STUDY00009021). This study has been registered with ClinicalTrials.gov (identifier NCT03301753).

### Method details

#### MILK study overview

Participant recruitment, clinical data, and milk sample collection for the Mothers and Infants LinKed for health (MILK) study have been described previously.^22^^,^^23^^,^^24^^,^^81^ Briefly, participants who intended to exclusively breastfeed were enrolled prenatally during healthy, uncomplicated pregnancies at the University of Minnesota in collaboration with HealthPartners Institute (Minneapolis, MN) or the University of Oklahoma Health Sciences Center. Recruited mothers were 21–45 years old, non-smokers, non-diabetic, and delivered singleton infants at full term (37 0/7–41 6/7 weeks gestation) with 10th–90th percentile birth weight on the WHO growth chart. No participants reported symptoms of mastitis or breast infection at the time of milk sample collection. Clinical data for each mother-infant dyad was collected from the delivering hospitals’ electronic health record and from electronic questionnaires at study visits at 1 and 6 months postpartum. Clinical study data were managed using REDCap electronic data capture tools hosted at the University of Minnesota. REDCap (Research Electronic Data Capture) is a secure, web-based software platform designed to support data capture for research studies. The data described in this manuscript comes from a subset of MILK Study mother/infant pairs who consented to maternal whole-genome sequencing, milk RNA sequencing, and microbiome assessment of infant fecal samples.

##### Gestational diabetes diagnosis

Gestational diabetes screening occurred between the 26th and 28th weeks of gestation by a 1-h blood glucose concentration after a 50 g oral glucose challenge test (OGCT). Women with OGCT levels greater than 130 g/dL then received a 3-h 100 g oral glucose tolerance test to confirm gestational diabetes. Gestational diabetes was diagnosed if a minimum of two out of four glucose level time point assessments were met or exceeded: 95 mg/dL (fasting), 180 mg/dL (1 h), 155 mg/dL (2 h), or 140 mg/dL (3 h).

##### Milk sample collection

Milk samples were collected at study visits at approximately 1 month postpartum, and infant fecal samples were collected at study visits at 1 and 6 months. Upon study visit arrival, participants fed their infants *ad libitum* from one or both breasts until infants were satisfied. Two hours following this feeding, milk was collected from the right breast using a hospital-grade electric breast pump (Medela Symphony; Medela, Inc., Zug, Switzerland), with expression ceasing when milk stopped flowing. Expressed milk volume and weight was recorded, milk was gently mixed, aliquots were made, and then stored at −80°C within 20 min of collection and kept at −80°C until thawed for RNA/DNA extraction.

#### Milk composition measurements

##### Human milk oligosaccharides

Concentrations of HMOs were quantified from 2 mL previously frozen whole milk aliquots as previously described.^82^ 19 HMOs were identified and quantified: 2′-fucosyllactose (2′FL), 3-fucosyllactose (3′FL), 3′-sialyllactose (3′SL), 6′-sialyllactose (6′SL), difucosyllactose (DFLac), difucosyllacto-N-hexaose (DFLNH), difucosyllacto-N-tetrose (DFLNT), disialyllacto-N-hexaose (DSLNH), disialyllacto-N-tetraose (DSLNT), fucodisialyllacto-N-hexaose (FDSLNH), fucosyllacto-N-hexaose (FLNH), lacto-N-fucopentaose (LNFP) I, LNFP II, LNFP III, lacto-N-hexaose (LNH), lacto-N-neotetraose (LNnT), lacto-N-tetrose (LNT), sialyl-lacto-N-tetraose b (LSTb), and sialyl-lacto-N-tetraose c (LSTc). Secretor milk was defined as having a 2′FL concentration that was greater than a natural, very low break in the data (Figure 3A). Weight-based concentrations were used for all statistical analyses (micrograms per milliliter). The sum of HMO concentrations was calculated as the total concentrations of the 19 measured HMOs. HMO concentrations were estimated over two batches, and HMO batch was included as a covariate in all analyses of HMO data.

##### Milk cytokines/nutrients/hormones

Milk fat was separated from the aqueous phase by centrifugation, and skim milk was assayed using commercially available immunoassay kits for insulin, glucose, leptin, CRP, and IL6 as previously described.^22^^,^^24^^,^^83^ These milk component assays were processed in 2–5 batches depending on the assay. Batch effects were corrected using an analysis of variance model with formula:

log(assayvalue)∼factor(batch)

using the ‘aov’ command in R. The residuals from this model, representing the batch-corrected values, were used in all downstream data analyses. There were not sample replicates across batches; original and corrected values are plotted in Figure S7.

##### Milk fat and lactose

Milk fat and lactose concentrations were assessed using mid-infrared spectrophotometry (Calais Milk Analyzer, North American Instruments, LLC, Lake Oswego, OR).^84^^,^^85^ Human milk samples were gradually thawed and then diluted with deionized water in a 1:1 dilution. Breastmilk control samples with standard macronutrient content were run prior to study sample testing to confirm instrument calibration. Samples were heated in a water bath until the samples reached 40°C and were mixed by gentle hand inversion for 2 min prior to analysis, per manufacturer instructions. Milk fat percent reliability was assessed in a random subset of 34 samples (17 duplicate samples) with an intraclass correlation coefficient (ICC) of 0.99, *p* < 0.001. Validity was assessed in a random subset of 30 samples against the gold standard Mojonnier method^83^ yielding a high cross-method ICC of 0.936, *p* < 0.001.

#### RNA extraction and sequencing

We extracted RNA from whole milk cell pellets to capture gene expression from both mammary epithelial cells and immune cells in milk. Previous studies that have performed bulk RNA-sequencing from human milk have used RNA extracted from the milk fat layer.^15^ This procedure enriches for milk fat globule RNA, which originates from mammary epithelial cells.^15^^,^^16^ Our approach allowed us to computationally estimate the contribution of different cell types to the milk transcriptomes, and explore genetic influences on gene expression that could be specific to the immune cells in milk, in addition to mammary epithelial cells.

Nucleic acid extractions and RNA-seq library preparation and sequencing was performed at the University of Minnesota Genomics Center (UMGC) in two batches (Table S1). In the first batch, frozen 2 mL whole milk aliquots from 245 milk samples were thawed and split in two, with each 1 mL half used for either RNA or DNA extraction. In the second batch, frozen 2 mL whole milk aliquots from 106 milk samples were thawed and the entire sample was used for RNA extraction. RNA was extracted from the cell pellet using the RNeasy Plus Universal HTP following the manufacturer’s instructions. We used the TakaraBio SMARTer Stranded Total RNA-seq Kit v2 - Pico Input Mammalian for RNA-seq library preparation. RNA libraries were sequenced on an Illumina NovaSeq 6000 S2 flow cell with 2 × 150 paired-end reads to a median depth of 36.8 million reads per sample. Sample-level details of RNA extraction and sequencing are in Table S1.

#### RNA-seq pre-processing and quantification

RNA-seq reads were trimmed with Trimmomatic and aligned with STAR^69^ v2.7.1a to the GRCh38 human reference genome. Gene-level quantification was performed with RNA-SeQC^70^ v2.3.4 using a Gencode v36 gene model annotation that was collapsed to a single transcript model per gene using a script provided by GTEx (“collapse_annotation.py” from https://github.com/broadinstitute/gtex-pipeline/tree/master/gene_model).

To assess the gene-level quantifications, TPM spearman correlations were calculated between each pair of samples with the ‘rcorr’ function from the ‘Hmisc’ R package.^86^ The first RNA-seq batch was sequenced in two pools (Table S1). Two samples that had poor quality in the first RNA-seq batch were re-run in the second RNA-seq batch (using an additional aliquot from the same original milk sample). We included only the replicate from the second batch for downstream analyses (Table S1). Samples with fewer than 10,000 genes detected were removed. There were five participants that contributed two milk samples, from two separate pregnancies. We included only one milk sample from each of these participants in our analyses, leaving 316 milk transcriptomes from 316 different participants (Table S1).

To explore technical sources of variation in our gene expression data, we performed a principal-component analysis of all 316 milk transcriptomes (Figure S1). We used the thinCounts function in edgeR to downsample each milk sample to 3,491,080 reads (the fewest reads in any one sample). We took the resulting count matrix as a DESeq2 object and performed a variance stabilizing transformation (VST). We then selected the 1000 most variable genes from the VST matrix, and performed principal-component analysis in R with the ‘prcomp’ function. Examining correlations between the PCs and quality control metrics of RNA extraction, library preparation, or sequencing, we selected five covariates to include in our differential gene expression analysis (below): batch, RIN, RNA concentration, number of genes detected, and mean 3′ bias (Figure S1). The ‘batch’ categorical variable had 3 levels representing the two sequencing pools of batch 1 and the single pool of batch 2 (Table S1; Figure S1).

#### Whole-genome sequencing and quality control

DNA was extracted from the cell pellet using the QIAamp 96 DNA Blood Kit at UMGC following the manufacturer’s instructions. Low-pass whole genome sequencing (WGS) at ∼1x and genotype imputation was performed by Gencove. Gencove’s low-pass WGS and imputation provides comparable or improved accuracy and variant discovery to array-based genotyping.^87^^,^^88^ 173 milk samples successfully underwent WGS and imputation from the original 1 mL aliquot DNA extraction. 72 samples had insufficient DNA extracted from the initial 1 mL sample, or failed Gencove’s quality control. Of these 72 samples, 62 had an additional 15 mL frozen aliquot that was shipped to Gencove and DNA was extracted using a mag Nucleic Acid Purification Kit (Biosearch Technologies), and ∼1x low-pass WGS was performed. 11 of these samples failed Gencove’s quality control and 51 samples successfully underwent WGS and imputation, resulting in 224 samples with genotype information. Finally, we submitted a third batch of 38 additional samples with 15 mL frozen aliquots to Gencove for DNA extraction and WGS as with the 15 mL aliquots above. 35 of these passed Gencove’s QC pipeline, resulting in a total of 259 samples with genotype information. Of the 19 total samples that failed Gencove’s QC pipeline, 1 failed the minimum bases sequenced and 18 failed the contamination metric (i.e., contamination by DNA from another sample of the same species, likely due to cross-sample contamination upstream of sequencing). 8 participants contributed 2 milk samples (from 2 separate pregnancies), and we included only one sample per participant in our analyses, leaving 251 unique individuals with genotype information. Sample-level details of extraction and sequencing are in Table S1. BCFtools^72^ was used to combine all VCFs into a BCF file for all individuals, filtering for minor allele frequency >1% and maximum missing genotypes of 5%. A genetic relatedness matrix was generated with the PLINK^73^ (v1.90b6.10) ‘--make-rel’ command, and one individual from pairs with relatedness coefficient >0.05 were pruned, leaving 230 individuals for genetic analyses.

To compare our genotypes to a well-defined population sample, we utilized the 1000 Genomes (1KG) 30x coverage whole genome sequencing dataset.^80^ VCF files containing genotypes for 2,504 participants were downloaded from https://www.internationalgenome.org/data-portal/data-collection/30x-grch38. We used BCFtools to combine all 1KG VCFs into a single BCF file, filtering for minor allele frequency >1% and maximum missing genotypes of 5%. We then used the BCFtools command ‘merge’ to create a single BCF file containing both the 1KG and milk study genotypes, filtering for genotypes missing in >5% of samples, thus removing variants absent in our milk study which comprised ∼8% of samples in the combined dataset. Genetic principal components (PCs) were calculated with PLINK using 902,579 SNPs with minor allele frequency >1% after pruning for linkage disequilibrium (PLINK command ‘—indep-pairwise 200 100 0.5’). The milk study participants mainly clustered with the European ancestry 1KG samples (Figure S2), in agreement with the genetic ancestry proportion estimates provided by Gencove, with only 19 of 230 individuals with estimated European ancestry <95% (Figure S3). We selected the first 3 genetic PCs to use as covariates in eQTL mapping.

We checked for sample swaps by performing genotype calling from RNA-seq reads aligned to chromosome 2 using ‘bcftools mpileup’, and using ‘bcftools gtcheck’ to compare genotypes from RNA-seq to Gencove variant calls from low-pass WGS.^72^ We did not detect any sample swaps: for all samples included in eQTL analysis, the DNA sample with matching sample ID had the lowest average concordance, compared to all DNA samples with a different sample ID (Figure S4).

### Quantification and statistical analysis

#### Comparison of milk transcriptomes to GTEx

We downloaded gene-level counts for GTEx samples from the GTEx portal (dataset GTEx_Analysis_2017-06-05_v8_RNASeQCv1.1.9_gene_reads.gct.gz). We filtered to only female GTEx samples, removed tissues with fewer than 19 remaining samples, and then selected 19 random samples for each tissue. We filtered to genes that were detected in both datasets after filtering genes with count 0 across all GTEx & milk samples, leaving 30,468 genes. We then used the thinCounts function in edgeR to downsample each GTEx and milk sample to 5 million read counts. We took the resulting count matrix as a DESeq2 object and performed variance stabilizing transformation (VST). We then took the VST matrix of only GTEx samples, selected the 1000 most variable genes, and performed principal-component analysis in R with the ‘prcomp’ function. We then projected the milk samples onto the PCA scatterplots by calculating 19 random milk sample’s values from the GTEx-only PCA to generate Figures 1A and S5.

To compare TPM values across milk and GTEx samples (Figure 1B), we downloaded gene-level TPM values from the GTEx portal (GTEx_Analysis_2017-06-05_v8_RNASeQCv1.1.9_gene_tpm.gct.gz). We filtered to include only female GTEx samples and filtered to protein-coding genes (as annotated in EnsDb.Hsapiens.v86) and removed histone genes. Our RNA library preparation kit (TakaraBio SMARTer Stranded Total RNA-Seq Kit v2 - Pico Input Mammalian) did not include polyA selection and histone gene mRNAs are not polyadenylated, resulting in higher detection of histone mRNAs in our data than in GTEx. We then rescaled the TPM for each GTEx and milk sample to again sum to 1 million and calculated each gene’s median TPM across a tissue type.

#### Correlations between milk gene expression and maternal/infant traits

We used edgeR^74^ to test for correlations between milk gene expression and maternal/milk traits, including all tested traits and technical covariates. Included traits were: Milk CRP concentration, milk glucose concentration, milk IL-6 protein concentration, milk insulin concentration, milk leptin concentration, milk volume expressed, gestational diabetes status, gestational weight gain, maternal pre-pregnancy BMI, maternal age, and parity (*N* = 269 milk samples with no missing data that were included in this analysis; Table S2). We also performed differential gene expression for two macronutrient traits (milk fat % and milk lactose %) separately, as these traits had the smallest sample size, and no individuals with gestational diabetes also had these measurements. Thus, we tested for gene expression for these traits including all other traits except gestational diabetes status as covariates on a smaller sample size (*N* = 171). We scaled each trait to a mean of zero and standard deviation of one, except binary traits (gestational diabetes status) and parity, for which we use the integer number of previous births. The count matrix and metadata were loaded into an edgeR object and the “filterByExpr” was used to remove lowly expressed genes, leaving 12,006 genes (or 12,332 genes for milk fat/lactose). We then used the ‘estimateDisp’ function on a design matrix regressing gene expression across all traits. This model accounted for potential confounding technical effects, including batch, RIN, RNA concentration, number of genes detected, and mean 3′ bias, by including them as covariates. We then used ‘glmQLFit’ to fit a quasi-likelihood negative binomial generalized log-linear model to the count matrix and design model, and ‘glmQLFTest’ to perform a quasi-likelihood F-test testing for the relationship between each gene against each tested trait. This model was selected for its handling of the over-dispersion in RNA-seq count data and type I error control.^89^^,^^90^ We used Benjamin-Hochberg correction of *p* values across all 12,006 genes (or 12,332 for fat/lactose) by 13 traits.

To assess the impact of RNA quality (as measured by RIN) on our differential gene expression results, we ran the same analysis on each trait in the top and bottom half of samples separately. Gestational diabetes status was excluded from this analysis because few samples with gestational diabetes were in the bottom half by RIN (only *N* = 5 samples with GDM). For the five traits with at least ten differentially expressed genes identified in the low RIN score subset (q value < 10%; milk glucose, IL-6, lactose, volume expressed, and parity), we tested for a correlation between the log fold-change estimates between the low and high RIN sample subsets for those genes. For all five traits there was a significant correlation (*p* < 0.01, r > 0.8 [except lactose]; Figure S8). Considering all genes, not just those significantly differentially expressed, there was a significant positive correlation between the top and bottom RIN subsets for all traits that had at least 50 differentially expressed genes in the full sample (Table S4). Thus, we moved forward with gene ontology enrichment for those traits with at least 50 differentially expressed genes.

We tested for gene ontology enrichment of significant genes (q value < 10%) for each trait using the R package topGO,^75^ with all tested genes as the background gene list. We used the ‘resultFisher’ function to run a classic Fisher’s exact test for each gene ontology, and used a Benjamini-Hochberg correction^91^ for all ontologies (*N* = 14,119) across the 7 traits with at least 50 significant genes (milk glucose, milk IL-6, milk insulin, milk volume expressed, gestational diabetes status, milk lactose %, parity; 98,833 tests). We report pathways with q value < 10%, fewer than 500 annotated genes, and an overlap of more than 5 genes with the significantly associated gene list for each trait (Table S5). All 7 traits had enriched ontologies that met these criteria.

To test for in interaction between maternal obesity status and the 6 traits with at least 50 significant differentially expressed genes (milk IL-6, milk glucose, milk insulin, milk lactose, parity, milk volume) with their association with milk gene expression, we filtered the 269 participants included in differential gene expression above into two categories based on pre-pregnancy BMI: ‘normal weight’ (*N* = 121, 18.5 ≤ BMI < 25) or ‘obese’ (*N* = 69, BMI ≥ 30). For milk lactose, after filtering individuals with missing data as described above, there were *N* = 78 ‘normal weight’ and *N* = 38 ‘obese’. Gestational diabetes was excluded from the interaction analysis because there were only 3 individuals with gestational diabetes in the ‘normal weight’ category. We then repeated the analysis as with the gene-wise model in the full sample above, but replacing BMI with this normal/obese categorical variable and including an interaction term between obesity status and the milk composition trait. Only gene/trait pairs with a significant correlation in the original analysis without an interaction term (q value < 10%) were tested. The interaction term *p* values were corrected across all included gene/trait pairs (4,525 tests) using a Benjamin-Hochberg correction (Table S6).

#### Examination of PER2 expression and milk traits

Circadian rhythm genes were defined as those in KEGG pathway ‘hsa04710’. To test if the time of day of the milk sample collection study visit explained the relationship between *PER2* expression and expressed milk volume, we transformed the time of the study visit into a quantitative variable with the R package ‘lubridate’.^92^ *PER2* expression values from a variance-stabilizing transformation of the sample-by-gene count matrix in DESeq2^71^ were used, including sample RNA mass and RIN as covariates. Regression models were calculated with ‘lm’ in R. Study time of day was correlated with *PER2* expression in a linear regression (*p* = 0.02), but not with milk volume expressed (B = −0.03, *p* = 0.4) We then ran the following linear models:

*PER2* expression ∼ milk volume + [technical covariates].

*PER2* expression ∼ milk volume + time of study visit + [technical covariates].

The same technical covariates included in differential gene expression testing were included here (batch, number of genes detected, RIN, RNA concentration, mean 3′ bias). These two linear models were compared by an F-test via the ‘anova’ command in R to test if adding the time of study visit term to the model provided a better fit to the data. This test (*p* = 0.06) suggested that adding the time of study visit variable did not provide a substantially better fit to the data. We used the ‘check_model’ function from R package ‘performance’^93^ to ensure that these models fit the linear regression model assumptions (Figure S8).

#### Deconvolution of bulk transcriptomes with bisque

Raw gene counts (MIT_Milk_Study_Raw_counts.txt.gz) and metadata (MIT_milk_study_metadata.csv.gz) were downloaded for the Nyquist et al. study^17^ from the Broad Insitute Single Cell Portal (https://singlecell.broadinstitute.org/single_cell/study/SCP1671/cellular-and-transcriptional-diversity-over-the-course-of-human-lactation) on 6/3/2022. Count data was filtered to keep just one sample per participant, requiring samples to have been collected >14 days and <3 months postpartum, leaving 10 samples. The count matrix and associated metadata was then formatted as a Bioconductor ‘ExpressionSet’ object, combining the two macrophage cell type annotations from Nyquist et al. into one cell type called just “Macrophage” and resulting in 8 cell type annotations. The milk gene-level count data was then loaded into an ExpressionSet object and Cell type deconvolution was run with the R package “BisqueRNA” and the function ‘ReferenceBasedDecomposition’, with parameters “markers = NULL” and “use.overlap = F”. Bisque^27^ used 19,387 genes present in both the bulk and single-cell expression sets. To generate the heatmap in Figure 1F, for each of the 8 cell types, sample cell type proportion estimates were regressed against all 8 traits (gestational diabetes status, gestational weight gain, maternal pre-pregnancy BMI, milk glucose, milk IL-6, milk insulin, milk volume expressed, parity) and technical covariates (RNA concentration, RIN, sequencing batch, number of genes detected, and mean 3′ bias) using the ‘glm’ function in R. The coefficients plotted are the regression coefficients for each trait for a given cell type from this multiple regression model.

#### Milk eQTL analysis

Gene-level quantifications were filtered for the 230 unrelated individuals with RNA-seq and genotype data. Genes were filtered to retain those with ≥6 counts and and TPM >0.1 in at least 20% of samples, leaving 17,672 genes of the original 45,473. TPM quantifications were then rank-normalized with the ‘RankNorm’ function in R package RNOmni,^94^ and gene coordinates were added using annotations from R package ‘EnsDb.Hsapiens.v86’. Genes without coordinate annotations, mitochondrial, and Y chromosome genes were removed, leaving 17,302 genes used in eQTL analyses.

The APEX toolkit was used for *cis*-eQTL analysis (https://corbinq.github.io/apex/doc/).^76^ First, 50 latent factors from the gene expression matrix were calculated using command ‘apex factor’ with 10 iterations. cis eQTL analysis was run with the command ‘apex cis’ with 3 genetic PCs (calculated with 1000 Genomes samples, described above) and 45 gene expression latent factors as covariates. The 45 latent factors were correlated with batch and other quality control metrics of the RNA-seq data (Figure S10). We used APEX’s linear mixed model with a genetic relatedness matrix calculated as above in PLINK, and with distance to start site weighting for eGene *p* values (ACAT-dTSS). SNPs with minor allele frequency >1%, missing genotype information <5%, and within 1 Mb of the gene transcription start site were included. The command used was as follows:

apex cis --bcf [genotypes bcf file] --bed [gene expression bed file] --cov [genetic PCs + gene expr. LFs covariate file] --grm [genetic relatedness matrix] --prefix [output file prefix] --long --dtss-weight 0.00001.

APEX uses an aggregated Cauchy association test to calculate a gene-level *p* value, and can use the distance to TSS weighting to improve discovery power (parameter ‘--dtss-weight’ in the command above). eGene *p* values were adjusted for multiple tests using a Benjamini-Hochberg correction.^91^

To assess the impact of RNA quality (as measured by RIN) on our eQTL results, we ran the eQTL scan on the top and bottom half of samples by RIN separately, as well as a random subset of the same size (*N* = 115). eGene *p* values were strongly concordant across all pairs of subsets and the entire *N* = 230 sample (*p* < 2 × 10^−16^; Figure S11), but with larger *p* values in the sample subsets reflecting the reduced power of a smaller sample size. Thus, we concluded that the lower RIN score samples in our eQTL analysis improved our power and should be included.

Conditional analysis of milk eQTLs were also run in APEX using the same covariates (3 genetic PCs, 45 gene expression latent factors) and the ‘--stepwise' flag:

apex cis --bcf [genotypes bcf file] --bed [gene expression bed file] --cov [genetic PCs + gene expr. LFs covariate file] --prefix [output file prefix] --long --dtss-weight 0.00001 --stepwise.

#### Colocalization of milk and GTEx eQTLs

eQTL summary statistics for single tissues (∗.v8.allpairs.txt.gz), and gene eQTL summary (∗.v8.egenes.txt.gz) were downloaded from the GTEx portal (https://gtexportal.org/). For each gene with an eQTL in milk at q value < 5%, each GTEx tissue with a significant eQTL (q value < 5%) was identified, and colocalization between the milk and GTEx tissue performed with the *coloc* R package^37^^,^^77^: *cis*-eQTL summary statistics for milk and each GTEx tissue with an eGene were filtered for those present in both milk and GTEx, within 200 kilobases of a top SNP of any tissue, and effect estimates harmonized so the reference/alternative alleles matched. LD matrices for these SNPs were generated using PLINK’s ‘--r square’ function with our genotyping data and using the European ancestry subset of the 1000 Genomes dataset (*N* = 503). eQTL signals for each tissue were fine-mapped using the ‘runsusie’ command, using the milk study LD reference for milk eQTLs and the 1000 Genomes LD reference for GTEx tissues. Colocalization was run between milk and each GTEx tissue with the command ‘coloc.susie’^36^ with a prior probability of colocalization of p_12_ = 3.5 × 10^−5^. This prior was chosen to require a lower burden of evidence for colocalization than the default value in coloc (p_12_ = 1 × 10^−5^), as here we are most interested in identifying milk-specific eQTLs and analyses of the GTEx project has demonstrated that most eQTLs are shared across tissues.^95^ Coloc.susie tests for colocalization between each pair of fine-mapped signals between the two tissues, and thus there will be multiple tests if fine-mapping identifies more than one signal for a particular tissue/gene pair. Each colocalization test was designated as ‘colocalized’ if the ratio PP.H4/(PP.H4+PP.H3) > 0.8; as ‘not-colocalized’ if the ratio PP.H3/(PP.H4+PP.H3) > 0.8; and ‘ambiguous’ otherwise.

Each fine-mapped milk eQTL signal was designated as milk-specific if either of these criteria were met: (1) there were no GTEx tissues with a significant eQTL for the gene (q value < 5%), or (2) there were no tissues with an eQTL signal that colocalized with the milk signal, and at least 75% of tested tissues’ eQTLs were categorized as not-colocalized. Of the 2,790 milk eGenes, 18 did not have an eQTL in any GTEx tissue, 401 failed at fine-mapping either the milk or GTEx signals, 1,907 had all eQTL signals colocalize with a GTEx eQTL, and 464 had at least one milk-specific eQTL signal. Enrichment analysis of genes with milk-specific eQTLs (*N* = 482) or tissue-shared eQTLs was performed with the ‘enrichGO’ command from the R package ‘clusterProfiler’,^96^ using a background gene list of all tested milk genes (17,302 genes) with a minimum gene set size of 10 and maximum size of 250.

#### Overlap between milk eGenes and dairy cattle QTL

Cattle gene coordinates for ARS_UCD1.2 genome were downloaded from https://bovinegenome.elsiklab.missouri.edu/downloads/ARS-UCD1.2, filtered for mRNAs, and for each gene with multiple entries the entry with the largest region was retained. Dairy cattle QTL were downloaded from the animalQTLdb (https://www.animalgenome.org/cgi-bin/QTLdb/index) by selecting “All data by bp (on ARS_UCD1.2 in bed format)”.

For each of 4 milk-related traits, we selected QTL with the following trait labels: milk yield (Milk yield, 305-day milk yield, Average daily milk yield), milk somatic cell count (Somatic cell score, Somatic cell count), milk protein (Milk protein percentage, Milk protein yield, Milk protein content), and milk fat (Milk fat percentage, Milk fat yield, Milk fat content). To identify a smaller list of genes identified in QTL from multiple studies, as some of these traits’ QTL overlapped thousands of genes, we identified genes that overlapped at least 1 QTL for all 4 dairy cattle milk traits (*N* = 1,035 genes, Table S11).

To test for enrichment of milk-specific vs. tissue-shared eQTL genes in these lists, we filtered milk eGenes for those that were present in the dairy cattle genome annotation above and that had a milk-specific eQTL (*N* = 146) vs. only tissue-shared eQTLs (*N* = 591). We performed a two-sided Fisher’s exact test where the 2 × 2 contingency table axes were: (A) milk-specific vs. tissue-shared eGenes (from our human milk eQTL analysis), and (B) cattle QTL overlapping genes vs. cattle QTL nonoverlapping (from the gene lists identified above), using the ‘fisher.test’ command in R.

#### Comparison of milk and GTEx eQTL with mash

We applied Multivariate Adaptive Shrinkage (*mash*) using the mashR package^40^ to assess patterns of eQTL sharing across milk and GTEx eQTLs. *mash* is an empirical Bayesian method that utilizes the covariance structure across conditions (in this application, tissues) to identify tissue shared or unique eQTL. We first identified the 13,593 genes that had eQTL summary statistics across all GTEx tissues and milk, as summary statistics from all tissues are required to run *mash*. Then, following the analysis outlined at https://stephenslab.github.io/mashr/articles/eQTL_outline.html, we extracted a ‘random’ matrix of summary statistics for 48 GTEx tissues and milk for 10,000 random gene/variant pairs. The ‘strong’ matrix was defined as the variant effects from all tissues for (1) the variants with the lowest milk eQTL *p* value for the 2,261 milk eGenes in this dataset; and (2) for each GTEx tissue, the variants with the lowest *p* value for 1000 random eGenes for that tissue. In total the ‘strong’ matrix contains summary statistics for 42,677 gene/variant pairs across 48 GTEx tissues and milk. From these input data we (1) estimate correlation structure from the ‘random’ matrix; (2) estimate data-driven covariances from the ‘strong’ matrix; (3) fit the *mash* model on the ‘random’ matrix using the data-driven and canonical covariances; and (4) estimate posterior summaries for the ‘strong’ matrix, i.e., re-calibrated effect estimates and statistical significance for each gene/variant pair in each tissue (Table S12). Using the output posterior summaries, we then calculated the fraction of milk eQTL effects that were shared with each GTEx tissue using the default criteria in *mashR*: local false sign rate <0.05, same direction of effect, and effect estimates within a factor of 2. This proportion of shared milk eQTL is plotted for a subset of GTEx tissues in Figure 2E. These tissues were chosen to represent the full range of similarity/dissimilarity to milk while not displaying all tissues for clarity of presentation. Results for all tissues are shown in Figure S13.

#### Colocalization of milk eQTLs with breast cancer GWAS summary statistics

GWAS summary statistics from Zhang et al.^43^ (icogs_onco_gwas_meta_overall_breast_cancer_summary_level_statistics.txt.gz) were downloaded from the BCAC website (http://bcac.ccge.medschl.cam.ac.uk/). Coordinates were converted to hg38 with LiftOver, and the meta-analysis summary statistics for all breast cancers were used (column names ‘Beta.Meta’, ‘p.meta’, etc.). For each milk eGene, colocalization was performed if there was a breast cancer GWAS hit of *p* < 5 × 10^−8^ within the eQTL window (within 1 Mb of gene TSS). Breast cancer GWAS and milk eQTL summary statistics were filtered to variants within 200 kb of the smallest milk eQTL *p* value, and statistics harmonized so the reference/alternative alleles matched. An LD matrix for these variants was calculated using (1) our milk study data and (2) the European ancestry subset of the 1000 Genomes European reference (*N* = 503). The milk and breast cancer GWAS signals were fine-mapped using ‘runsusie’ in the *coloc* R package,^36^^,^^37^^,^^77^ using the milk LD reference for the milk eQTLs and the 1000 Genomes LD reference for the breast cancer signals. Colocalization was run with the command ‘coloc.susie’ with a prior probability of colocalization of p_12_ = 5 × 10^−6^. We chose this prior based on the recommendation in Wallace.^97^

#### Correlations between milk gene expression and oligosaccharides

HMOs were rank normalized within the 310 individuals with both gene expression and HMO data, using the ‘RankNorm’ function from R package ‘RNOmni’.^94^ For HMOs absent in non-secretors (2′FL and DFLac; Figure S21), we included only secretor individuals (*N* = 231). The following HMO categories were also calculated: the sum of all HMO concentrations, the sum of all sialylated HMO concentrations (DSLNH, DSLNT, FDSLNH, LSTb, LSTc, 3′SL, 6′SL), and the sum of all fucosylated HMO concentrations (DFLNH, DFLNT, FDSLNH, FLNH, LNFP-I, LNFP-II, LNFP-III, 3′FL, DFLac, 2′FL). These HMO category sums were rank normalized across all individuals.

We used edgeR^74^ to test for correlations between milk gene expression and HMO concentrations. The count matrix and metadata were loaded into an edgeR object and “filterByExpr” was used to remove lowly expressed genes, leaving 11,780 genes (or 11,695 genes for secretors only). For each HMO, we then used the ‘estimateDisp’ function on a design matrix regressing gene expression across HMO concentration, secretor status (except for when only secretors were included, i.e., 2′FL and DFLac), HMO batch, sequencing batch, RIN, RNA concentration, number of genes detected, and mean 3′ bias. We then used ‘glmQLFit’ to fit a quasi-likelihood negative binomial generalized log-linear model to the count matrix and design model, and ‘glmQLFTest’ to perform a quasi-likelihood F-test of each gene against each tested HMO. We used Benjamin-Hochberg^91^ correction of *p* values across all HMO-gene pairs.

We tested for gene ontology enrichment of significant genes (q value < 10%) for each trait using the R package topGO, with all tested genes as the background gene list. We used the ‘resultFisher’ function to run a Fisher’s exact test for each gene ontology, and used a Benjamini-Hochberg correction^80^ for all ontologies (*N* = 14,034) across the 15 HMOs/HMO categories with at least 50 significant genes (Table S16).

#### Genetic associations at milk eQTLs with milk oligosaccharides

The list of candidate genes to test for effects of milk eQTLs on HMO concentrations was downloaded from Supplementary Dataset 2 in Kellman et al.^48^ From this gene list, we identified 7 genes with significant eQTLs in our dataset (q value < 5%). To test for genetic associations between the lead variant identified by fine-mapping above (all 7 genes had only one signal detected) at each milk eGene and HMO concentrations using rank-normalized HMO concentrations. For 2′FL and DFLac, which were absent in non-secretors (Figure S21), we rank-normalized the concentrations within secretors and scaled concentrations in non-secretors to have mean −3 and s.d. 0.1, to avoid introducing variation that did not exist in non-secretors. We used ‘glm’ in R to fit a model with HMO concentrations as the outcome, including genotype, secretor status, HMO batch, and the first three genetic PCs as covariates:

HMO ∼ genotype + secretorStatus + HMO batch + PC1 + PC2 + PC3.

For models of HMOs vs. *FUT2* eQTL genotype, we excluded the secretor status term. Genotype vs. HMO concentration plots in Figures 3D and 3E show the residual HMO concentration after regressing out HMO batch and the first 3 genetic PCs. For Figure 3E, secretor status was also regressed out of the plotted FLNH concentrations.

To estimate the effect of modified milk gene expression on HMO concentrations, we used a Wald Ratio, which estimates the causal effect between an exposure (milk gene expression) and outcome (HMO concentration) by dividing a single genetic variant’s effect on outcome by the genetic effect on the exposure.^98^

#### Processing of infant fecal metagenomes

Infant fecal collection and storage, and metagenomic DNA extraction were described previously.^81^ Briefly, feces were collected from diapers either during study visits and frozen at −80°C immediately, or collected at home, stored in 2 mL cryovials with 600 μL RNALater (Ambion/Invitrogen, Carlsbad, CA), and stored at −80°C after shipping to the lab at the University of Minnesota. DNA was extracted using the PowerSoil kit (QIAGEN, Germantown, MD), eluted with 100 μL of the provided elution solution, and stored in microfuge tubes at −80°C.

Extracted DNA was used to construct libraries for metagenomics sequencing using the Illumina Nextera XT kit (Illumina, San Diego, CA, United States). Metagenomics libraries were then sequenced on an Illumina NovaSeq system (Illumina, San Diego, CA) using the S4 flow cell with the 2 × 150 bp paired end V4 chemistry kit by the University of Minnesota Genomics Center, achieving a sequencing depth of ∼4.5 million reads per sample.

Microbial taxon abundances were generated by first processing metagenomic fastq files with Shi7 version 1.0.1,^99^ which learns optimal quality control parameters from the data. Sequences were then trimmed, filtered by quality scores, and stitched per the learned parameters in Shi7. Sequences from all samples were multiplexed into a single fasta file for downstream processing. Processed sequences were aligned to reference databases using BURST version 0.99.7f,^78^ using a reference genome database generated from GTDB r95 (https://gtdb.ecogenomic.org/stats/r95). A 95% identity cutoff and forward/reverse complement flag were used. Resulting.b6 files were converted to reference and taxonomy tables using embalmulate^78^ with ‘GGtrim’ activated. To generate microbial pathway abundances, metagenomic sequences were run through the MetaPhlAn^79^ version 3.0.7 pipeline, with BowTie2^100^ version 2.4.2 64-bit, DIAMOND^101^ version 0.9.24, and MinPath^102^ version 1.5.

To generate the PCA of infant metagenomes in Figure 4A, data were filtered to include only taxa with relative abundance >0.001 in at least 10% of 1-month or 6-month samples. A centered log-ratio transformation was performed on the relative abundances of each sample, and principal components were calculated with the ‘prcomp’ command in R.

#### Sparse CCA of human milk transcriptomes and infant fecal metagenomes

Input datasets were prepared as follows.

##### Milk gene expression

To prepare gene expression data for this analysis, the sample-by-gene count matrix was loaded into DESeq2,^71^ filtered to keep only protein-coding genes with count > 0 in at least half the participants (14,905 genes), and transformed using the variance stabilizing transformation. After this transformation, the variance of each gene was calculated across all samples and genes in the lowest 25% variance were removed, leaving 9,421 genes.

##### Infant fecal metagenomes

Taxon abundances and pathway abundances from 1- and 6-month infant fecal samples were processed separately. The taxon relative abundance matrix was filtered to retain species-level taxa only, keeping only species with a relative abundance >1 × 10^−3^ in at least 10% of samples (92 species for 1-month and 82 species for 6-month samples). A centered log-ratio transformation was then performed on each sample’s relative abundances. For microbial pathways, species-specific and unclassified pathways were removed, leaving 241 pathways for 1-month and 216 pathways for 6-month samples. The species and pathway level information from both timepoints was then combined into one matrix.

Each dataset was filtered for the 146 individuals with both 1- and 6-month infant fecal metagenomes and 1-month milk gene expression. Sparse canonical correlation analysis (sparse CCA), to identify sparse components maximizing correlation between the milk gene expression and infant fecal metagenome datasets, and enrichment analyses of genes in each sparse component, were performed as previously described,^53^ using k = 15 components. Code was downloaded from https://github.com/blekhmanlab/host_gene_microbiome_interactions. Significance of the sparse components was calculated with leave-one-out cross-validation, and 12 components were retained at Benjamini-Hochberg q value < 10%. Pruning significant components whose scores across mother-infant pairs were correlated at Pearson’s r > 0.5 left 7 remaining sparse components (Figure 4B). Pathway enrichment was performed separately on positively weighted and negatively weighted genes for each component.

To generate network interaction plots between milk-expressed genes and infant fecal microbes identified in the sparse CCA analysis, for each significantly enriched pathway (q value < 10%) in a component, we (1) filtered for overlapping genes between the component and pathway; (2) generated a pairwise correlation matrix of mother-infant pairs’ trait values for those genes, the top 3 microbiome traits in the component with positive weights, and top 3 microbiome traits with negative weights; (3) pruned for correlations with Pearson’s r > 0.3 and *p* < 0.05; (4) generated a network plot from the pairwise correlation matrix using the ‘ggnetwork’ package in R.^103^

*B. infantis* growth rates were estimated using Compute PTR (CoPTR).^61^ We aligned the infant gut metagenomic shotgun reads to the *B. infantis* ATCC 15697 reference genome, downloaded from NCBI, using bowtie2 v2.2.4.^100^ We then used CoPTR to get coverage information for each mapped sample, filtering for samples with at least 75% coverage and at least 3000 mapped reads to the *B. infantis* genome. For samples that passed these filters, CoPTR was used to estimate the peak-to-trough ratio (PTR) from the coverage information, an estimate of the bacterial growth rate.

## Supplemental information

Document S1. Figures S1–S24 and Tables S2, S4, and S14

Table S1. Overview of study samples, related to STAR Methods Summary of samples used in each analysis, and sequencing metrics. study_id, unique parent/infant dyad ID; RNA_conc, concentration of RNA in milk sample (ng/μl); RIN, RNA Integrity Number; rna.batch, batch of RNA sequencing & extraction; RNAreadCount, number of RNA-seq reads; RNAuniqMapPct, percentage of uniquely mapping RNA reads (output from STAR); Genes Detected, number of genes with at least one read count; Mean 3′ bias, mean 3′ bias of RNA mapping (from rnaseqc); WGS_effective_coverage_min, Gencove effective coverage; WGS_fraction_contamination_max, Gencove estimated sample contamination; WGS_snps_min, Gencove number of called SNPs; dna.batch, batch for DNA sequencing; expr.incl: logical statement, was sample included in gene expression analyses; eqtl.incl: logical statement, was sample included in eQTL analyses; inf.fecal.1mo, if sample has 1-month infant fecal metagenomic data (logical); inf.fecal.6mo, if sample has 6-month infant fecal metagenomic data (logical); notes, empty except for 2 duplicate samples that were removed; sibling, study_id of sibling pairs (same parent, different child).

Table S3. Output of correlation analysis between maternal/milk traits and gene expression in milk in EdgeR, related to Figure 1 Only nominally significant (*p* < 0.05) associations are reported. logFC, log2 fold change in gene expression per unit 1 change in trait value; logCPM, log2 counts per million; F, quasi-likelihood F-test statistic; PValue, *p* value; gene_id, Ensembl gene ID of gene tested; gene_name, gene name of gene tested; FDR, Benjamini-Hochberg q value across all tested trait-gene pairs; trait, trait tested.

Table S5. Enrichment analysis in TopGO for significant genes in correlation analysis between maternal/milk traits and milk gene expression, related to Figure 1 GO.ID, Gene Ontology ID; Term, GO name; Annotated, number of annotated GO genes included in background gene list; Significant, number of GO genes significantly correlated with the tested trait (q value < 10%); Expected, expected number of significant genes in GO gene list; p, Fisher’ test *p* value; FDR, Benjamini-Hochberg q value corrected across all tested GO terms and traits; trait, tested trait.

Table S6. Interaction analysis between maternal/milk traits and maternal obesity in association with gene expression in milk in EdgeR, related to STAR Methods The reported statistics are for the interaction term between maternal obesity status (normal weight vs. obese) and the tested trait. logFC, log2 fold change in gene expression per unit 1 change in trait value; logCPM, log2 counts per million; F, quasi-likelihood F-test statistic; PValue, *p* value; trait, trait tested; gene_id, Ensembl gene ID of gene tested; gene_name, gene name of gene tested; FDR, Benjamini-Hochberg q value across all tested trait-gene pairs.

Table S7. Correlations between maternal/milk traits and estimated cell type proportions, related to Figure 1 Regression coefficients testing for correlations between maternal/milk traits and 8 Bisque-estimated cell type proportions, in a regression model including all tested traits and technical covariates (see STAR Methods). B, trait regression coefficient; SE, standard error of coefficient estimate; P, *p* value of coefficient estimate; cellType, cell type; trait, maternal/milk trait; FDR, Benjamini-Hochberg q value across all cell type/trait pairs.

Table S8. Summary of eQTL analysis across 17,302 tested genes, related to Figure 2 gene, Ensembl gene ID; gene_name, gene name; gene_biotype, gene biotype; n_samples, number of samples in eQTL analysis; n_cis_variants, number of nearby (cis) genetic variants tested for association with gene expression; egene_pval, gene-level aggregated Cauchy association test (ACAT) *p* value (see STAR Methods); FDR, Benjamini-Hochberg q value across all tested genes; milkSpecific: logical, was eQTL identified as milk-specific in GTEx eQTL colocalization analysis.

Table S9. Summary of conditional eQTL analysis, related to Figure 2 Each gene has one or more rows for conditionally independent *cis*-eQTL signals. gene, Ensembl gene ID; gene_name, gene name; nsignal, index of this signal out of the total number of signals for this gene; snp, index variant for this signal; rsID, variant ID; beta, SNP effect estimate; SE, standard error of SNP effect estimate; p_joint, *p* value from joint analysis; p_acat, cauchy aggregation test *p* value weighted by TSS; p_marginal, *p* value from single SNP association test; p_sequential, *p* value from stepwise regression.

Table S10. Summary of colocalization results comparing milk eQTLs to GTEx, related to Figure 2 Each row represents the output of coloc.susie for a fine-mapped milk eQTL lead variant, genes with multiple rows had more than one fine-mapped signal. gene, Ensembl gene ID; gene_name, gene name; milk.hit, fine-mapped lead variant for the milk eQTL; milk.hit.rsID, variant ID for fine-mapped milk lead variant; nTiss, number of GTEx tissues with a significant eGene for this gene (q value < 0.05); nTests, number of colocalization tests for this milk signal, may be larger than the number of tissues if any tissue had more than one fine-mapped signal; nColoc, number of colocalization tests that colocalized.

Table S11. List of genes overlapping QTLs for all four dairy cattle milk traits, related to Figure 2 Used to test for enrichment of dairy cattle milk trait QTL genes in milk-specific vs. tissue-shared eQTLs.

Table S12. Output of the mash model of milk and GTEx eQTL effects, related to Figure 2 Posterior estimates for lead variants of 2,093 milk eQTL with local false sign rate <0.05 are reported. gene: Ensembl gene ID; varID: variant chrom_position_ref_alt; rsID: variant ID; ∗_pm: posterior mean for each tissue; ∗_lfsr: local false sign rate (measure of significance) for each tissue.

Table S13. Breast cancer GWAS loci tested for colocalization with milk eQTLs, related to Figure 2 Each row represents a single colocalization result, if a milk eQTL or breast cancer association had multiple signals, there may be multiple rows per gene. milk_egene, Ensembl gene ID; milk_egene_name, gene name; nsnps, number of SNPs in both milk eQTL & GWAS summary statistics used in coloc analysis; milk_variant, fine-mapped top SNP for milk eQTL; milk_variant_ID, rsID of fine-mapped top SNP for milk eQTL; milk_credible_set_index, index of credible set for milk eQTL credible set in this colocalization; brca_variant, fine-mapped top SNP for breast cancer association; brca_variant_ID, rsID of fine-mapped top SNP for breast cancer association; brca_credible_set_index, index of credible set for breast cancer credible set in this colocalization; PP.H0.abf, coloc posterior probability of hypothesis 0 (neither trait has causal variant); PP.H1.abf, coloc posterior probability of hypothesis 1 (only milk eQTL has a causal variant); PP.H2.abf, coloc posterior probability of hypothesis 2 (only breast cancer has a causal variant); PP.H3.abf, coloc posterior probability of hypothesis 3 (both traits have causal variant, not a shared causal variant); PP.H4.abf, coloc posterior probability of hypothesis 4 (both traits have a shared causal variant); Beesley/Ferreira/Fachal/Zhang logical, was this gene connected to a GWAS locus in the cited publication.

Table S15. Output of correlation analysis between HMO concentrations and gene expression in milk in EdgeR, related to Figure 3 Only nominally significant (*p* < 0.05) associations are reported. logFC, log2 fold change in gene expression per unit 1 change in HMO concentration; logCPM, log2 counts per million; F, quasi-likelihood F-test statistic; PValue, *p* value; HMO, HMO or HMO category tested; gene_id, Ensembl gene ID of gene tested; gene_name, gene name of gene tested; FDR, Benjamini-Hochberg q value across all tested HMO-gene pairs.

Table S16. Output of enrichment analysis in TopGO for significant genes in correlation analysis between HMOs and milk gene expression, related to Figure 3 GO.ID, Gene Ontology ID; Term, GO name; Annotated, number of annotated GO genes included in background gene list; Significant, number of GO genes significantly correlated with the tested trait (q value < 10%); Expected, expected number of significant genes in GO gene list; HMO, tested HMO/HMO category; P, Fisher’ test *p* value; FDR, Benjamini-Hochberg q value corrected across all tested GO terms and HMOs/HMO categories.

Table S17. Glycolsyltransferase genes with milk eQTLs, related to Figure 3 Starting from a list of 54 candidate glycosyltransferase genes,^48^ seven genes had significant milk eQTLs in our data. gene_name, gene name; gene, Ensembl gene ID; egene_pval, eQTL gene-level *p* value if gene was included in eQTL analysis; FDR, eQTL gene-level q value if gene was included in eQTL analysis; top_snp, chromosome, base position, reference, and alternative alleles for variant with the smallest eQTL *p* value; rsID, rsID for variant in top_snp; snp_beta, estimated effect on gene expression for top_snp; snp_se, standard error of SNP effect on gene expression for top_snp; snp_pval, *p* value of effect on gene expression for top_snp.

Table S18. Associations between milk eQTLs and HMO concentrations, related to Figure 3 Genetic associations between candidate HMO gene eQTL tag genetic variations and HMO concentrations, and Wald Ratio estimates of the effect of genetically modified gene expression on HMO concentration. gene, Ensembl gene ID; gene_name, gene name; rsID, rsID for variant in top_snp; top_snp, chromosome, base position, reference, and alternative alleles for variant with the smallest eQTL *p* value; HMO, tested HMO; N, number of individuals with genotype data at this variant and HMO data for association analysis; ga.est, estimated SNP effect on HMO concentration; ga.se, standard error of SNP effect on HMO concentration; ga.p, *p* value of SNP effect on HMO concentration; ga.q, Benjamini-Hochberg q value of SNP effect on HMO concentration; wr.b, Wald ratio estimate of the effect of genetically modified gene expression on HMO concentration; wr.se, standard error of Wald ratio effect estimate; wr.p, *p* value of Wald ratio effect estimate.

Table S19. Results of sparse CCA integrating milk transcriptomes and infant fecal metagenomes, related to Figure 4 Sparse CCA output weights for identified sparse components containing milk-expressed genes and infant fecal microbial taxa or pathways. feature, feature name; weight, weight of feature in sparse component; type, is feature a milk gene (gene) or infant fecal microbial trait (microbe); component, the sparse component this feature weight is for.

Table S20. Results of pathway enrichment analysis on milk-expressed genes identified in components of sparse CCA output, related to Figure 4 pathway, pathway name; genes_in_pathway, number of genes in pathway annotation; genes_in_path_and_BG, number of genes in pathway and background set; genes_of_interest, number of genes in component gene set; genes_of_interest_in_pathway, number of genes in component gene set in pathway; gene_names, names of overlapping genes in pathway; odds_ratio, odds ratio of overlap; p_val, *p* value of overlap; type, were postively (pos.wt) or negatively (neg.wt) weighted genes tested; component, the sparse component this enrichment test is for (matches components in Table S19); p_adj, Benjamini-Hochberg corrected *p* value.

Table S21. Pairwise Pearson correlations between expression levels of JAK-STAT pathway genes in milk, infant fecal *B. infantis* growth rate and relative abundance at 1 month, infant fecal *E. coli* abundance at 6 months, milk IL-6 concentration, milk glucose concentration, gestational diabetes status, and milk LSTc (HMO) concentration, related to Figure 4 t1, trait 1; t2, trait 2; r, correlation coefficient; P, correlation *p* value; N, sample size of correlation estimate; FDR, Benjamini-Hochberg q value.

Document S2. Article plus supplemental information
