# Supplementary material for: Human milk variation is shaped by maternal genetics and impacts the infant gut microbiome
Source: Cell Genom. 2024 Sep 11;4(10):100638. doi: 10.1016/j.xgen.2024.100638 (PMC11602576; doi:10.1016/j.xgen.2024.100638)
Supplement: Document S1. Figures S1–S24 and Tables S2, S4, and S14 [file mmc1.pdf]

Cell Genomics, Volume *4*

Supplemental information

Human milk variation is shaped by maternal genetics and impacts the infant gut microbiome

Kelsey E. Johnson, Timothy Heisel, Mattea Allert, Annalee Fürst, Nikhila Yerabandi, Dan Knights, Katherine M. Jacobs, Eric F. Lock, Lars Bode, David A. Fields, Michael C. Rudolph, Cheryl A. Gale, Frank W. Albert, Ellen W. Demerath, and Ran Blekhman

Supplemental material for:

"Human milk variation is shaped by maternal genetics and impacts the infant gut microbiome"
K.E. Johnson, et al. *Cell Genomics* 2024

Figure S1. 2
Figure S2. 3
Figure S3. 4
Figure S4. 5
Figure S5. 6
Figure S6. 7
Figure S7. 8
Figure S8. 9
Figure S9. 10
Figure S10. 11
Figure S11. 12
Figure S12. 13
Figure S13. 14
Figure S14. 15
Figure S15. 16
Figure S16. 17
Figure S17. 18
Figure S18. 19
Figure S19. 20
Figure S20. 21
Figure S21. 22
Figure S22. 23
Figure S23. 24
Figure S24. 25
Table S2. 26
Table S4. 27
Table S14. 28

Figure S1.

Technical factors correlated with human milk transcriptomes, Related to Figure 1. Principal components of milk transcriptomes are plotted, with the left hand column plotting PC1 vs. PC2 and the right column PC3 vs. PC4. Each point represents a milk sample. In each row the points are colored by a different metric, designated the by color legend on the right of each row.

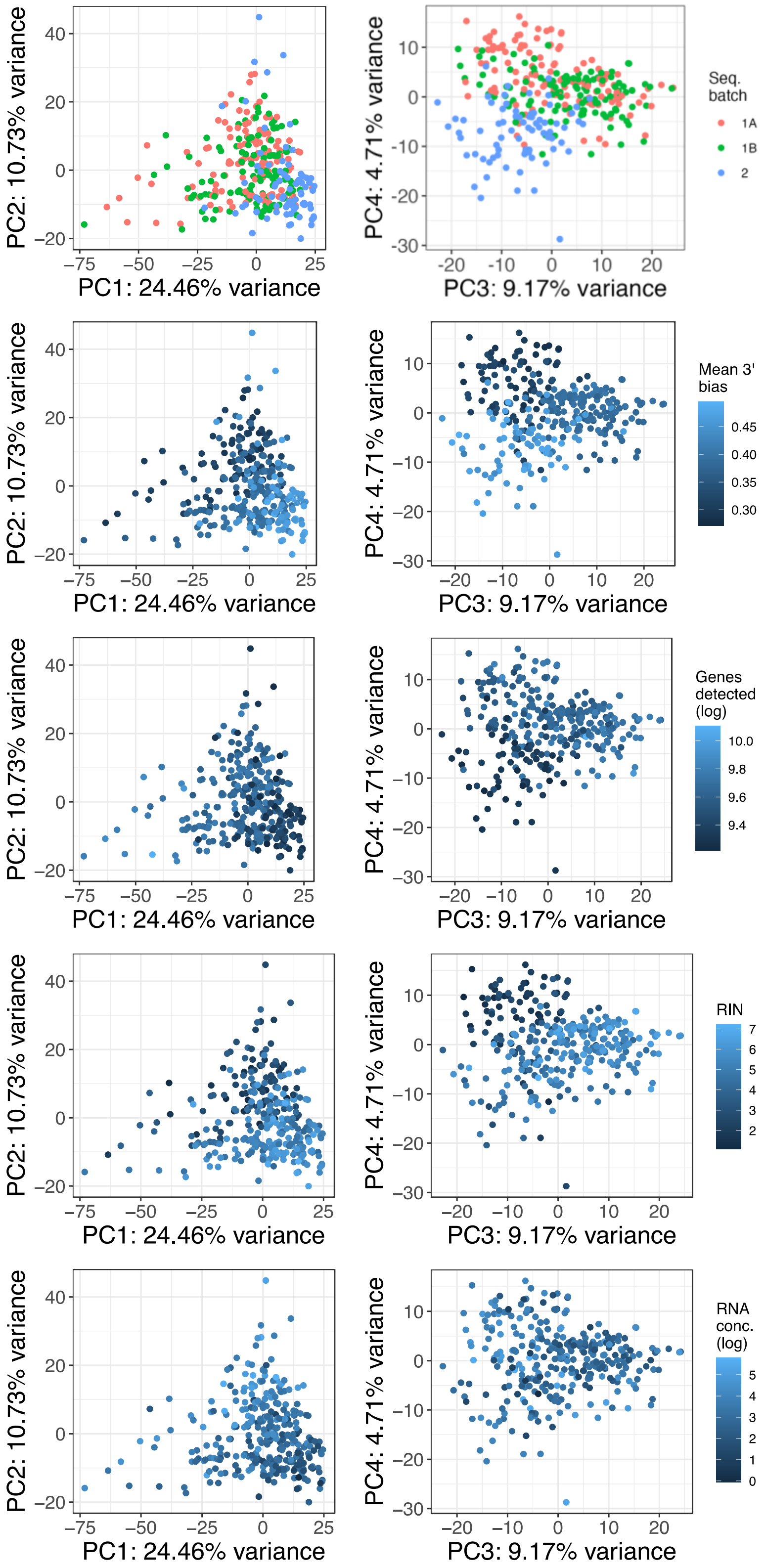

Figure S2.

Principal components of study participants genotypes, Related to STAR Methods. Principal components analysis of low-pass whole genome sequencing data from this study (black points) with reference samples from the 1000 Genomes Project (points colored by continental group). AFR: Africa, AMR: America, EAS: East Asia, EUR: Europe, SAS: South Asia.

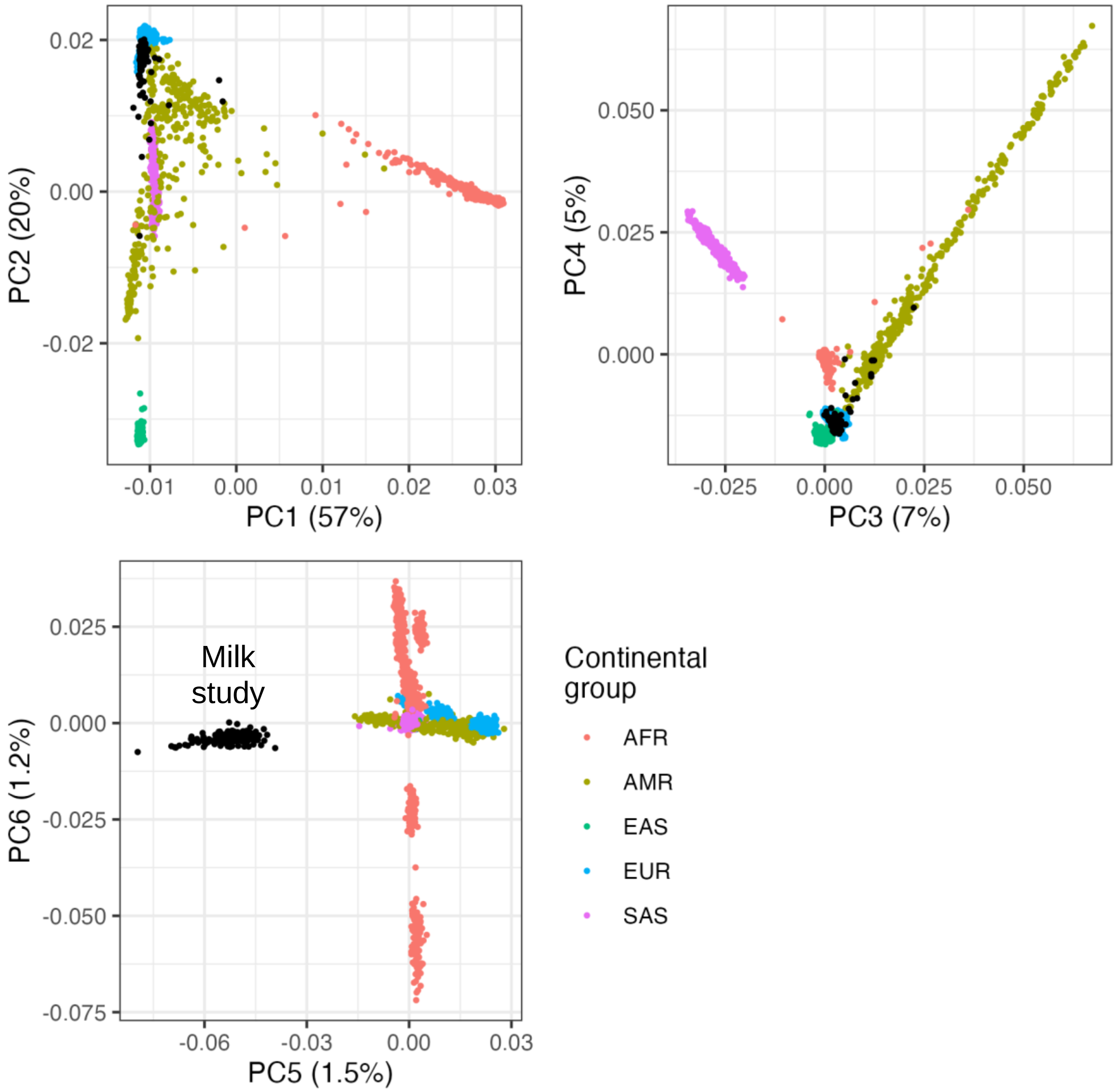

Figure S3.

Genetic ancestry estimates of study participants, Related to STAR Methods. Distributions of genetic ancestry estimates for individuals included in the eQTL analysis. Within each panel, representing a continental ancestry group, is a histogram displaying the distribution of estimated ancestry proportions for that group for all samples. e.g. all samples have an estimated European ancestry proportion >0.4, with the majority ~1; while no samples have estimated African ancestry proportion > 0.3.

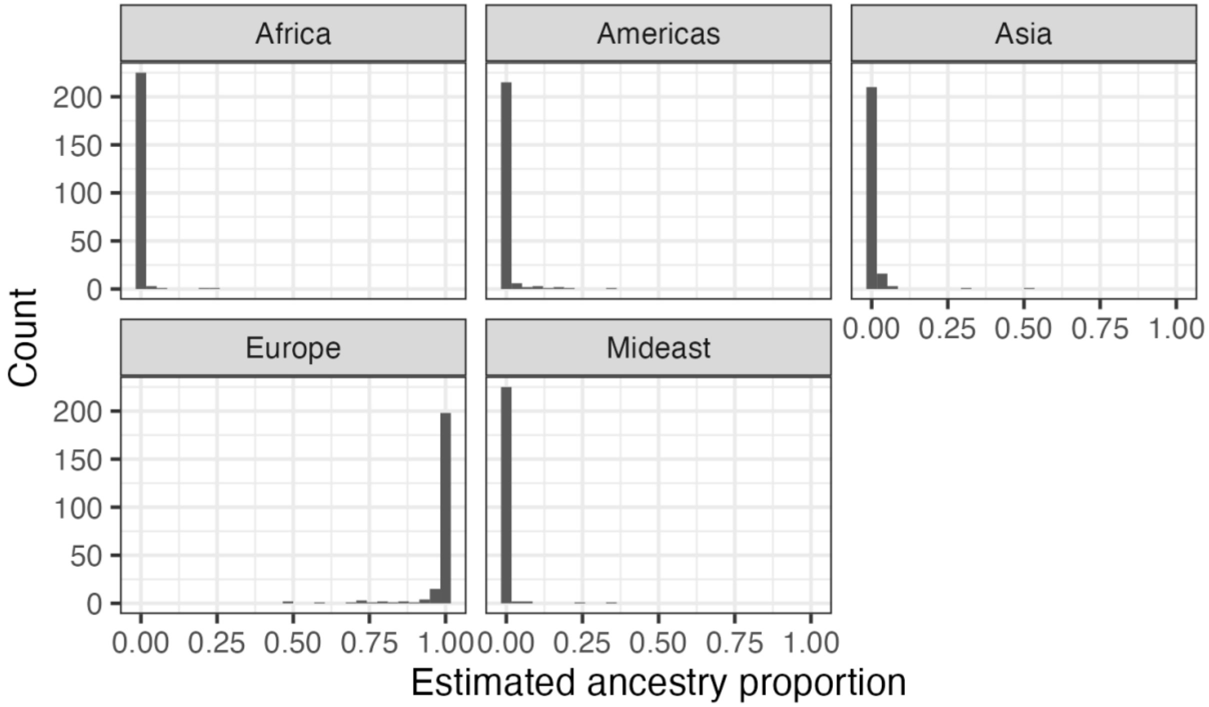

Figure S4.

Checking for sample mix-ups between milk RNA and DNA sequencing data, Related to STAR Methods. Distribution of discordance between genotypes estimated from RNA and DNA samples. Each dot represents a milk sample ID, with the x-axis showing the discordance between genotype calls using either the RNA or DNA sequencing data from the same sample ID. The y-axis is the minimum discord between that sample ID's RNA sample and any DNA sample. All points are above the x=y line (dashed line), showing that the DNA sample with the matching sample ID always had the most similar genotype calls for each RNA sample, and that there were no sample label mix-ups. Points are colored by RNA sequencing pool/batch ('rna.batch').

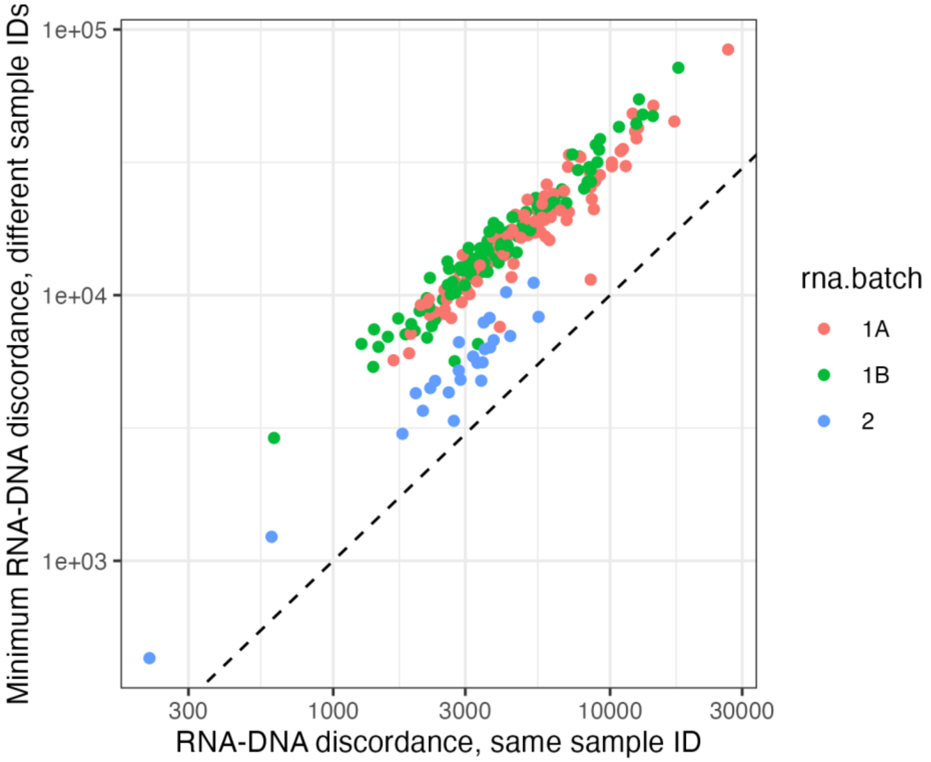

Distribution of discordance between genotypes estimated from RNA and DNA samples. Each dot represents a milk sample ID, with the x-axis showing the discordance between genotype calls using either the RNA or DNA sequencing data from the same sample ID. The y-axis is the minimum discord between that sample ID's RNA sample and any DNA sample. All points are above the x=y line (dashed line), showing that the DNA sample with the matching sample ID always had the most similar genotype calls for each RNA sample, and that there were no sample label mix-ups. Points are colored by RNA sequencing pool/batch ('rna.batch').

Figure S5.

Principal components analysis of milk samples with GTEx tissues, Related to Figure 1. Principal component analysis of transcriptomes from a subset of GTEx tissues and milk. PCs were calculated using the 1000 most variable genes within GTEx, then milk samples were projected onto the GTEx samples.

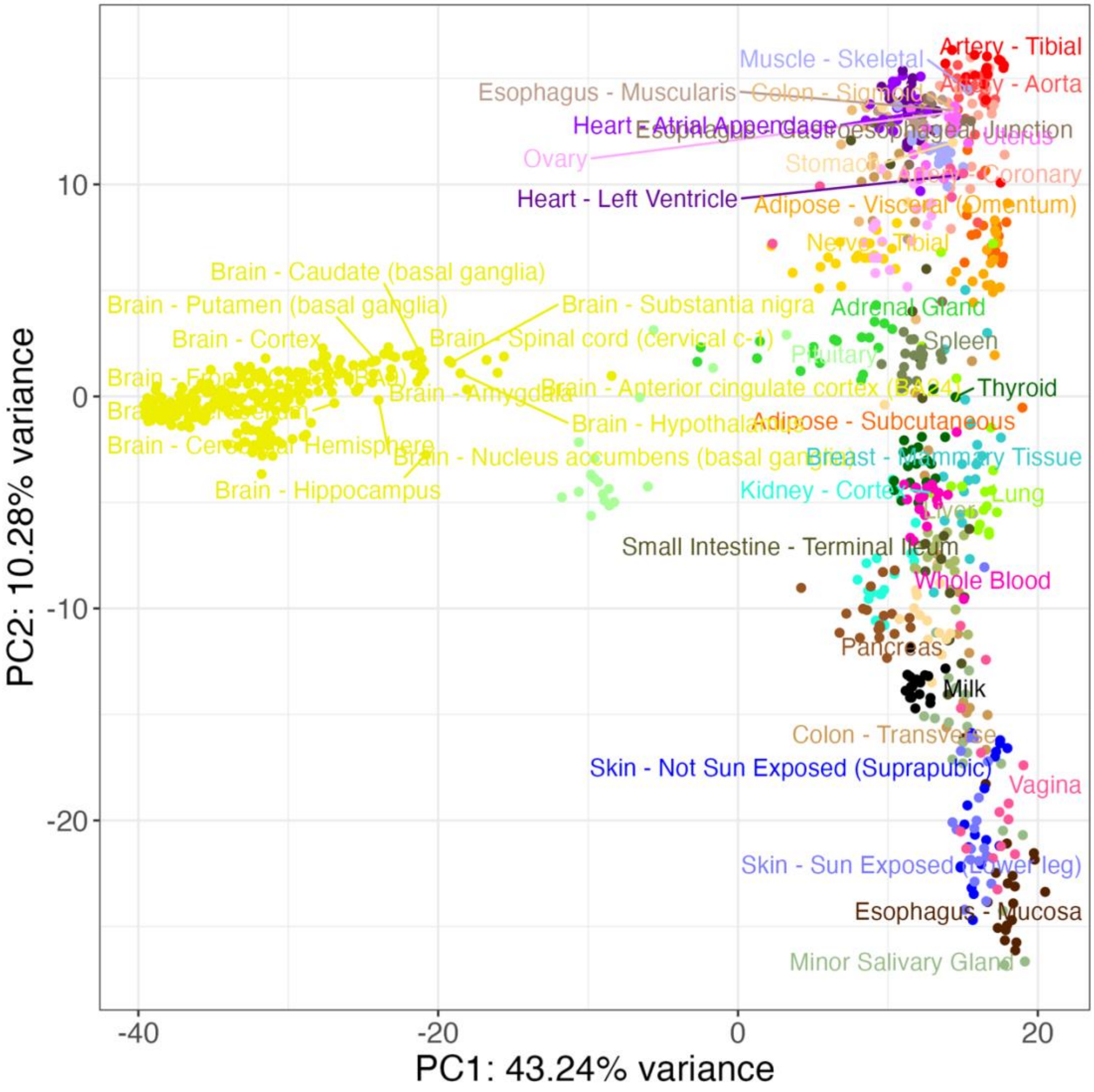

Figure S6.

Correlations between maternal and milk traits in the MILK study, Related to STAR Methods. Spearman correlations between the 13 maternal/milk traits tested for relationships with milk gene expression. An asterisk signifies q-value < 0.05, correcting for all pairwise comparisons. Individuals with gestational diabetes did not have milk macronutrient values available, thus correlations between those traits were not estimated (indicated by gray boxes).

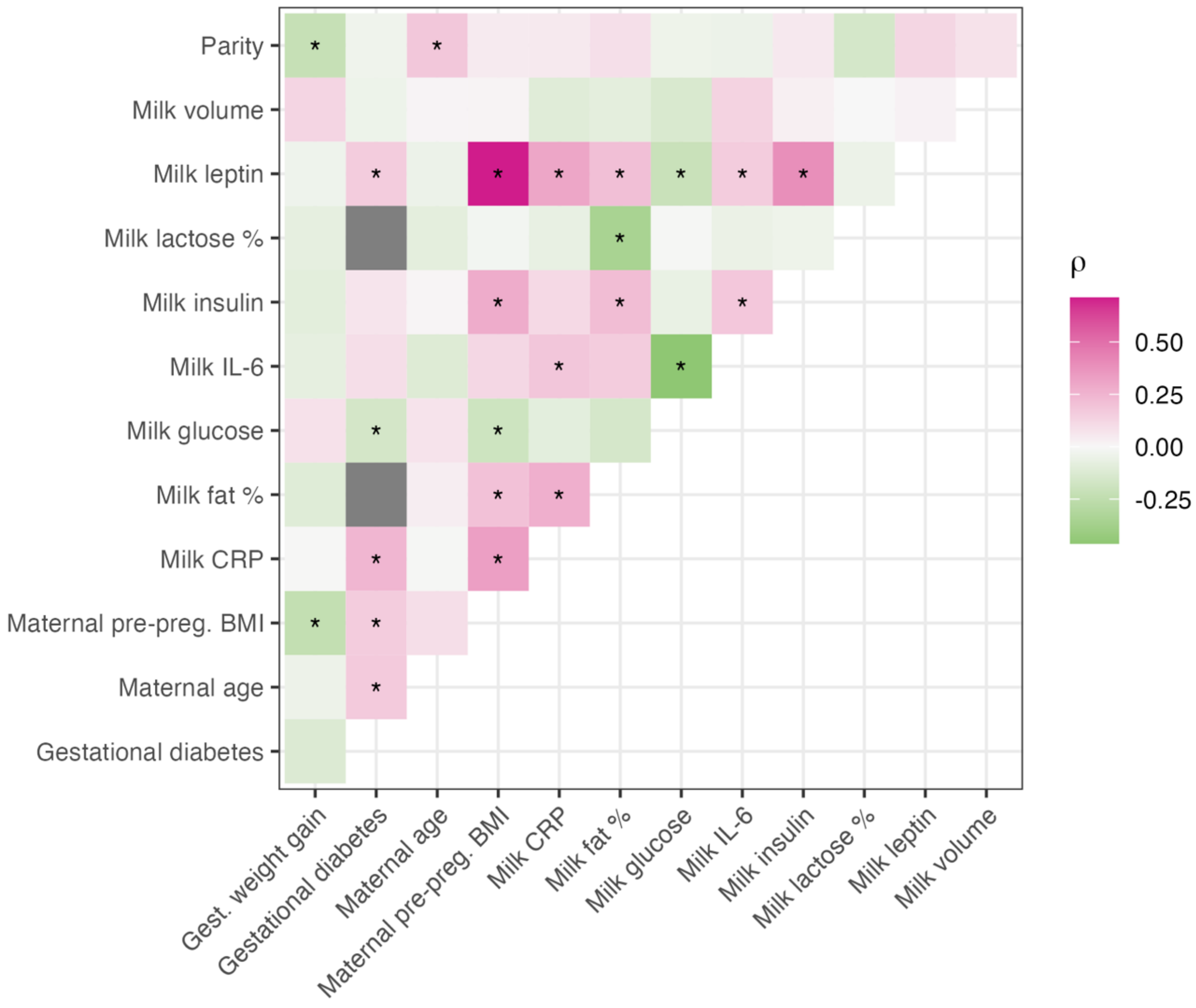

Figure S7.

Milk composition lab values before and after batch correction, Related to STAR Methods. Milk composition lab values before and after batch correction. The left hand column are the original values after a log transformation, and the right hand column are the batch-corrected values. Each point is a milk sample, plotted by lab assay batch along the x-axis and lab value on the y-axis.

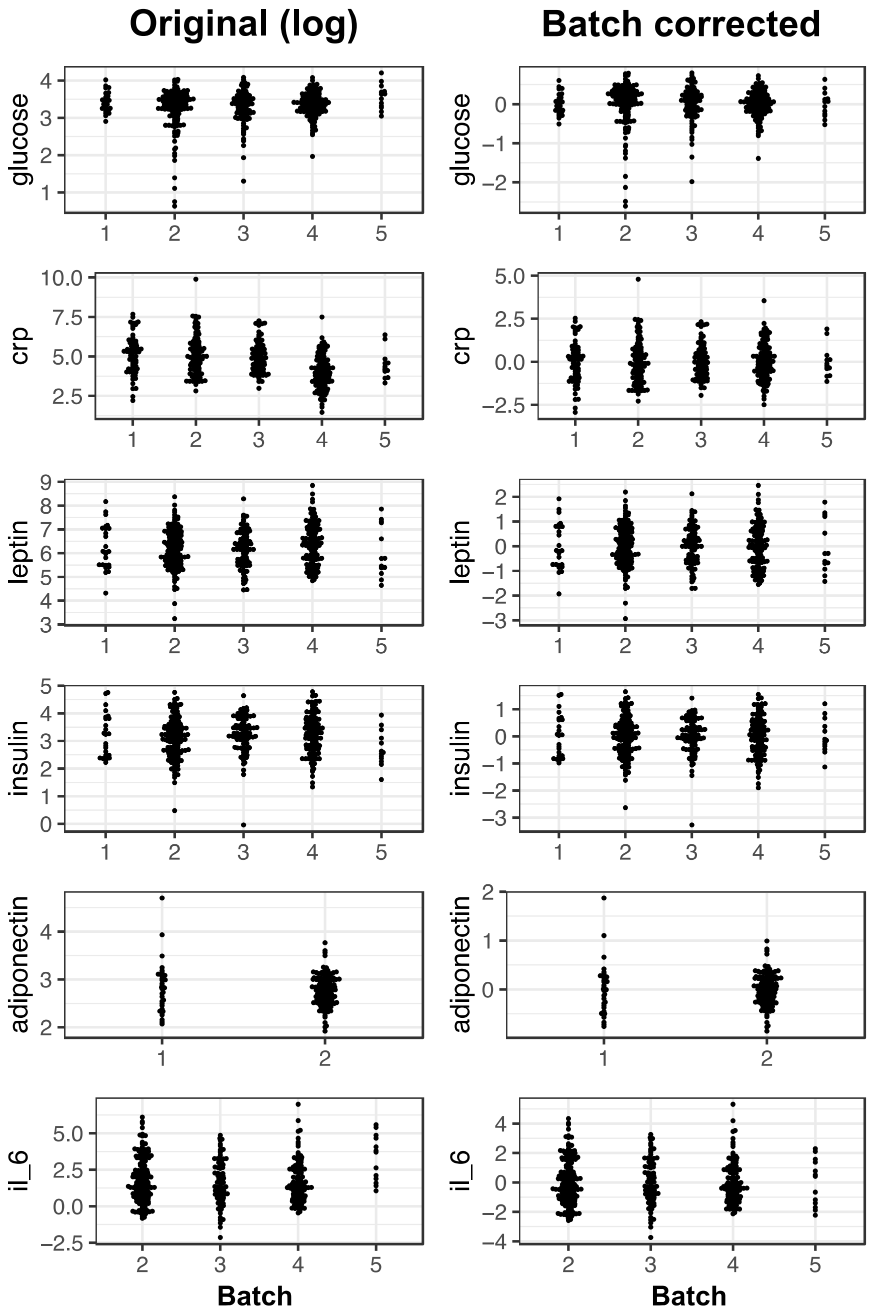

Figure S8.

Robustness of trait-gene expression correlations to RNA Integrity Number (RIN), Related to STAR Methods. Pearson correlations between estimated log fold-change (logFC) from trait-gene expression correlations performed using the top (x-axis) or bottom (y-axis) half of samples by RIN. Each point represents a gene, and genes were included if they were significantly correlated with the trait (q-value <10%) in the analysis with the bottom half of RIN samples. The blue line represents a linear regression line and confidence interval for the plotted points.

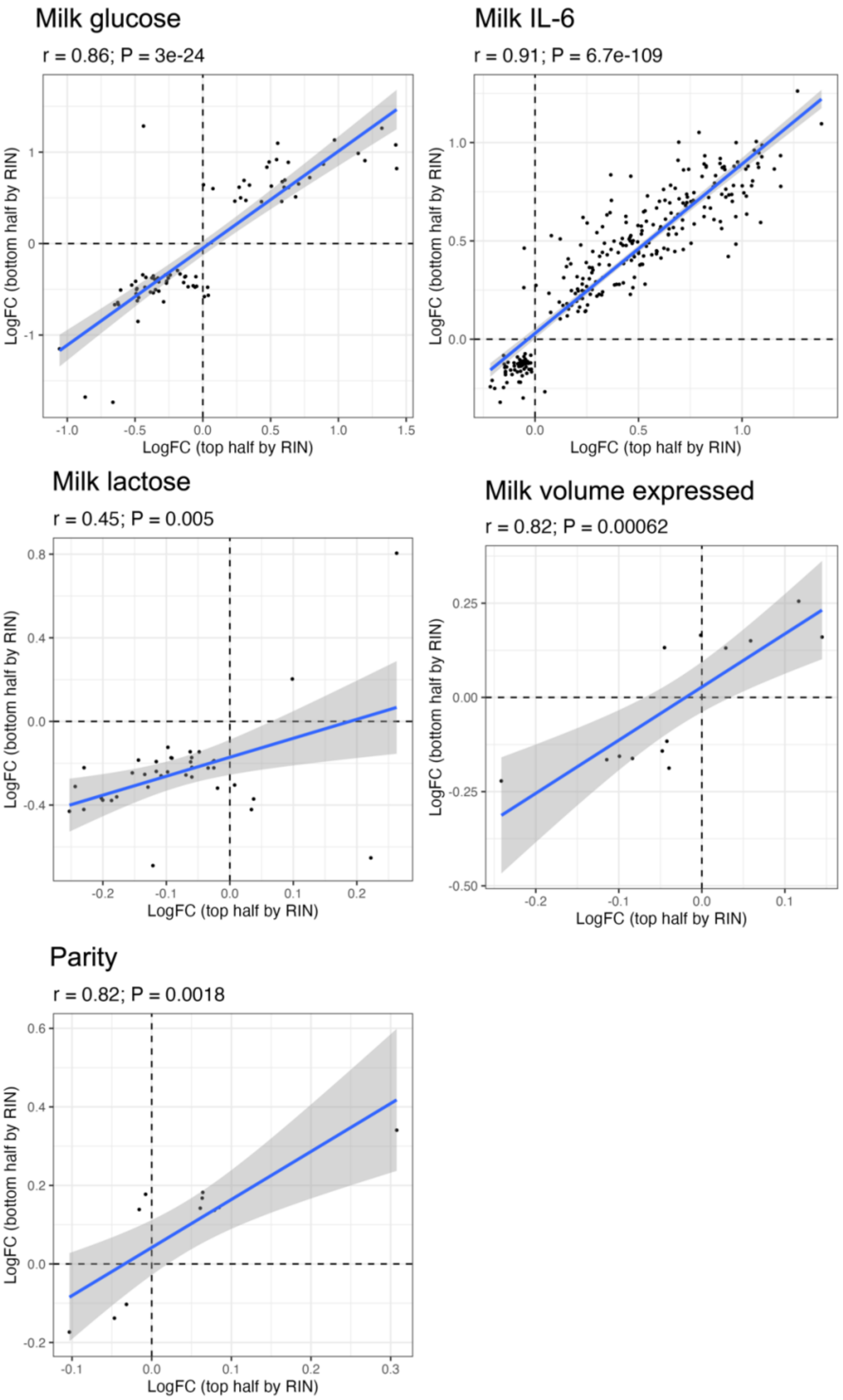

Figure S9.

Model checks for comparison of *PER2* expression to sample collection time and milk volume, Related to STAR Methods. Model assumption checks for the multivariate linear regression model used to confirm that adding sample collection time of day did not improve the model testing for a correlation between *PER2* expression and milk volume expressed. This plot was generated using ‘the check_model’ function from R package ‘performance’. The model fits these checks reasonably well, with some deviations at the tails of the distribution.

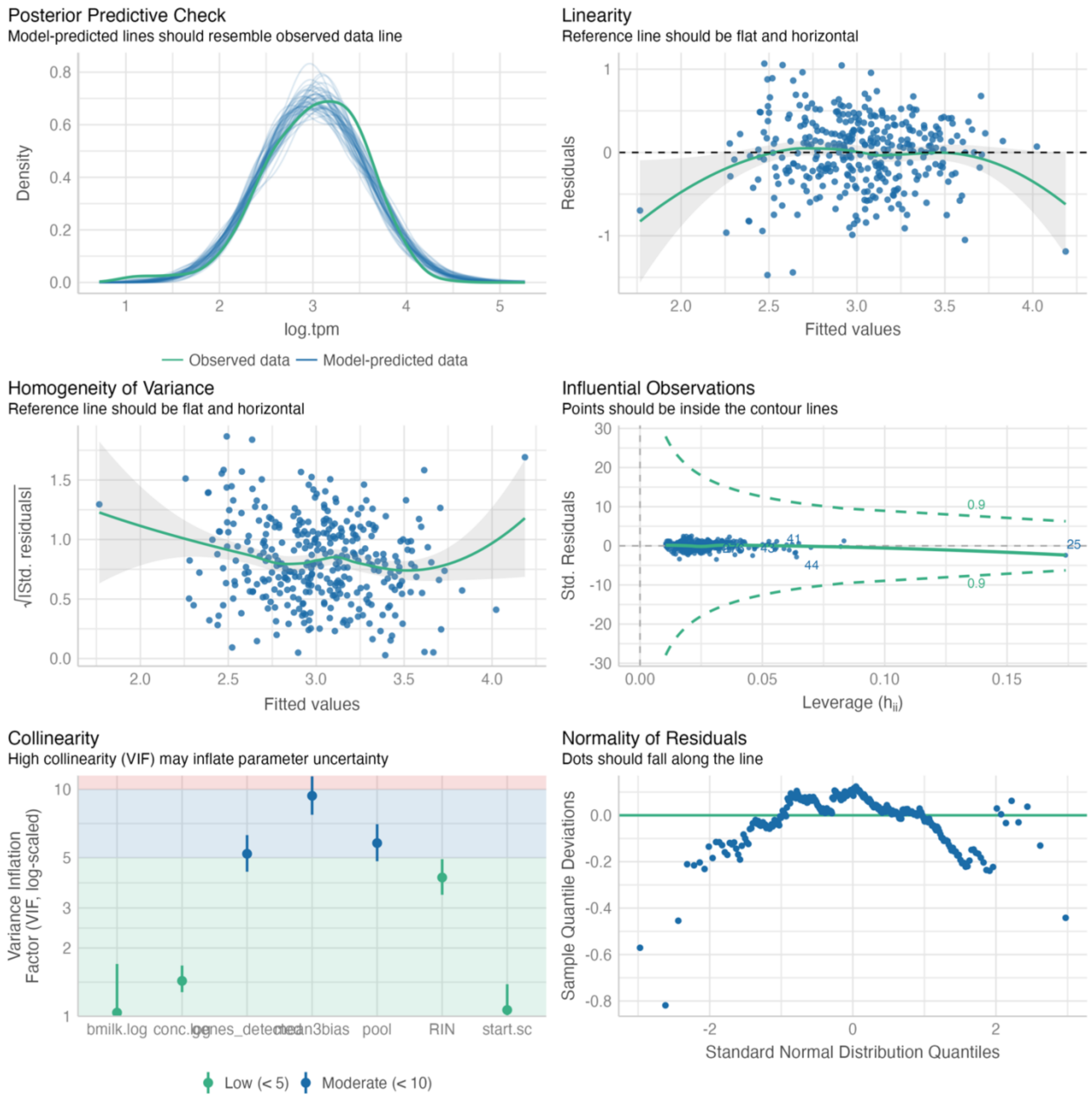

Figure S10.

Correlations between milk and maternal traits or RNA-seq technical and inferred latent factors of milk transcriptomes, Related to STAR Methods. Correlations between milk/maternal traits and RNA-seq quality control metrics (x-axis) and the latent factors of gene expression utilized as covariates in eQTL mapping. Colored boxes are plotted for trait/factor pairs with correlation p-value<0.05. rho = Spearman correlation coefficient.

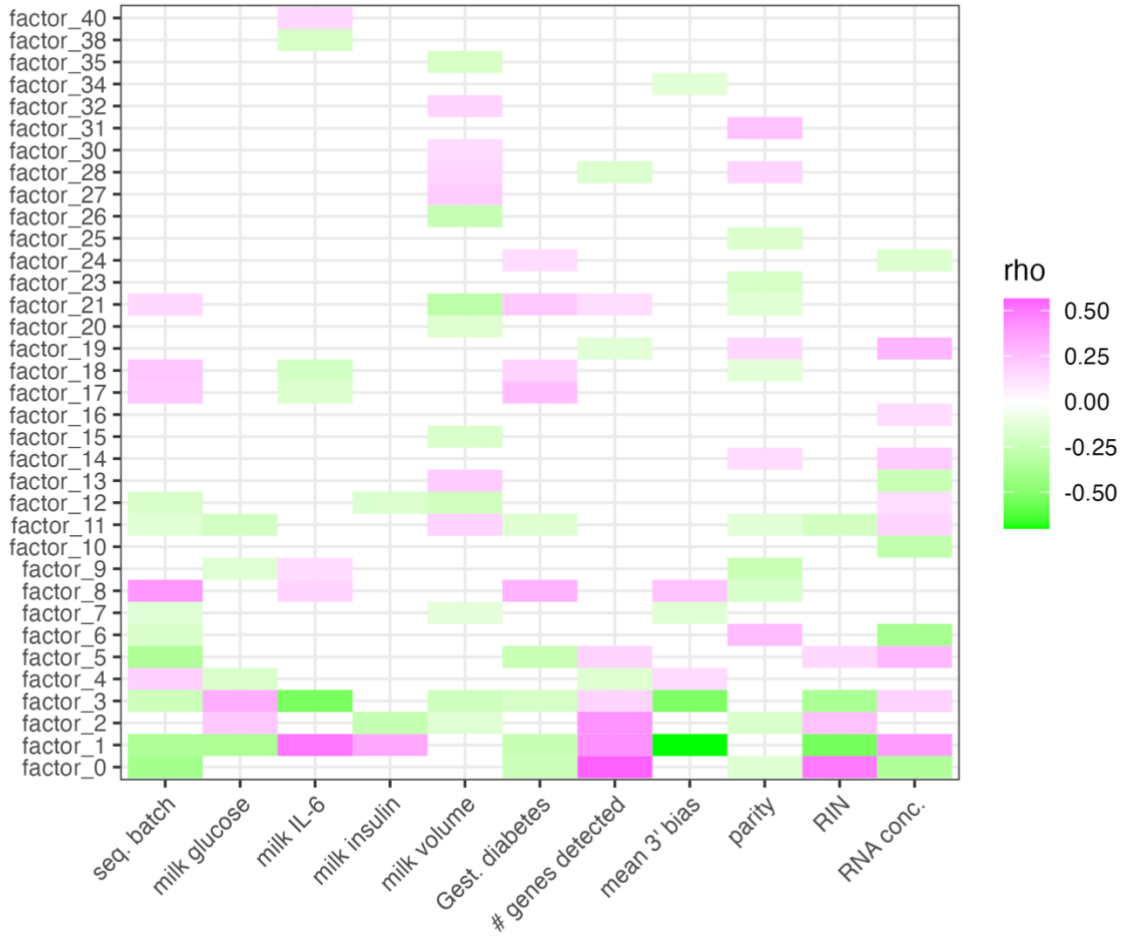

Figure S11.

Robustness of eQTL detection to sample RNA Integrity Number (RIN), Related to STAR Methods.
Comparison of eGene q-values when the eQTL scan was performed with the bottom half of samples by RIN, top half of samples by RIN, a random subset of half the samples, or all N=230 samples. Each point represents a gene, and the pink line is the identity line. For all pairwise comparisons, there was a significant correlation. The final three plots comparing the full sample to the sample subsets demonstrate the loss of power by reducing sample size, as the points diverge from the identity line in pink.

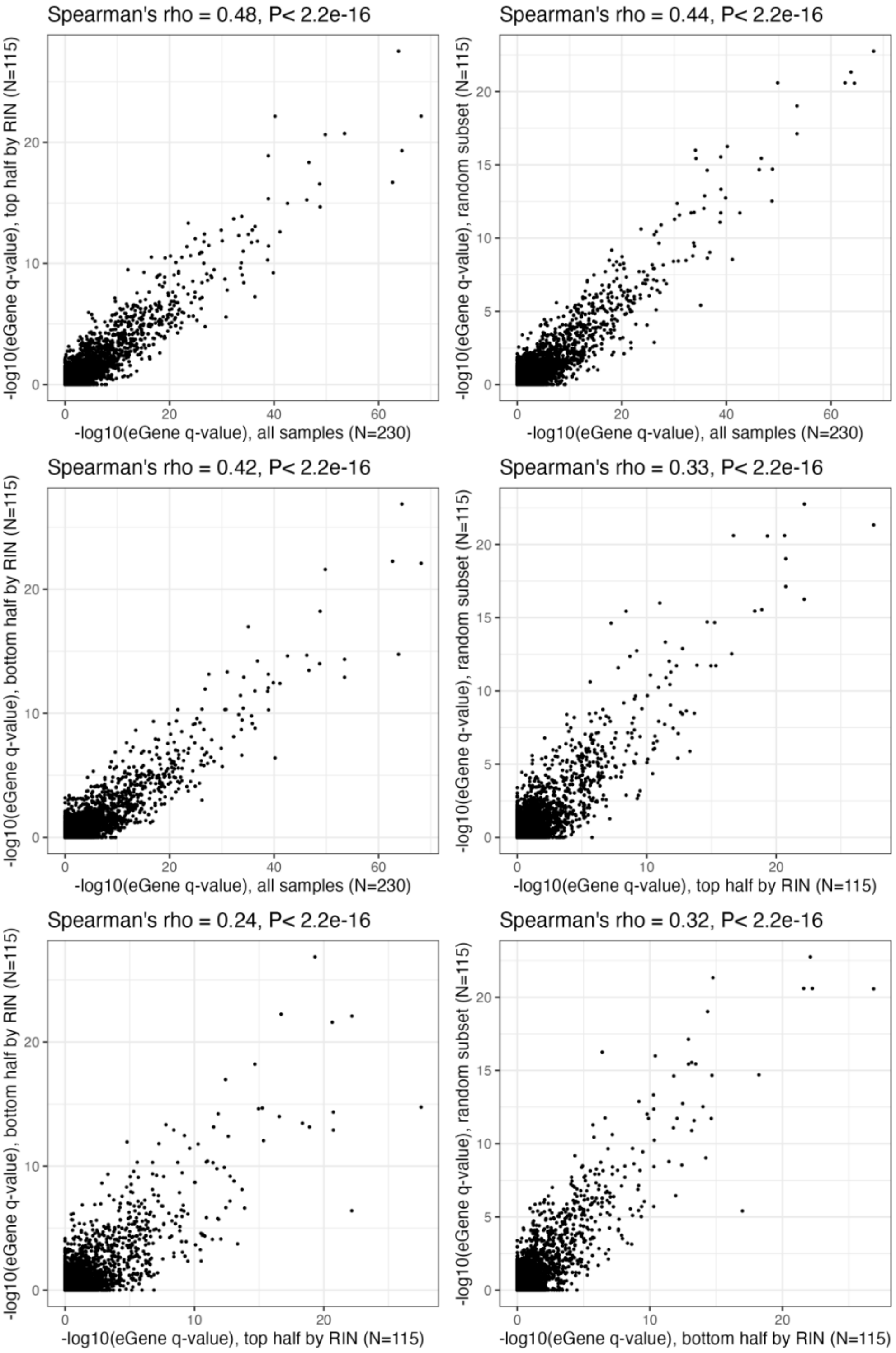

Figure S12.

Fraction of eGenes detected when subsampling data by RIN, Related to STAR Methods. Fraction of tested genes identified as eGenes (i.e., with eGene q-value < 0.05) for subsamples of our dataset by top/bottom half of RIN score (N=115), a random subset with N=115, or the entire sample (N=230). There was no significant difference in the proportion of eGenes between the random subset and bottom half by RIN (P=0.78, Pearson's chi-squared two-sided test). All other pairwise comparisons were significant (P<0.005).

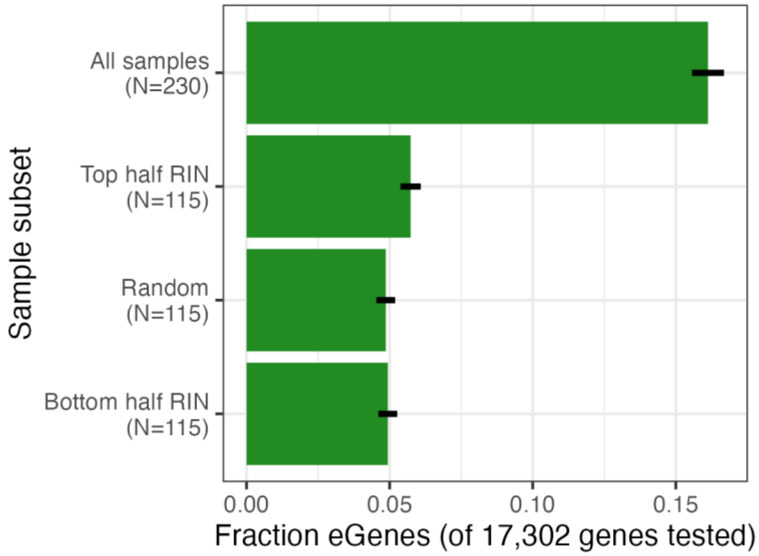

Figure S13.

Proportion of shared eQTLs between milk and GTEx tissues, Related to Figure 2. For each GTEx tissue, the figure shows the proportion of milk eQTLs that were shared with the tissue based on the output of *mash*.

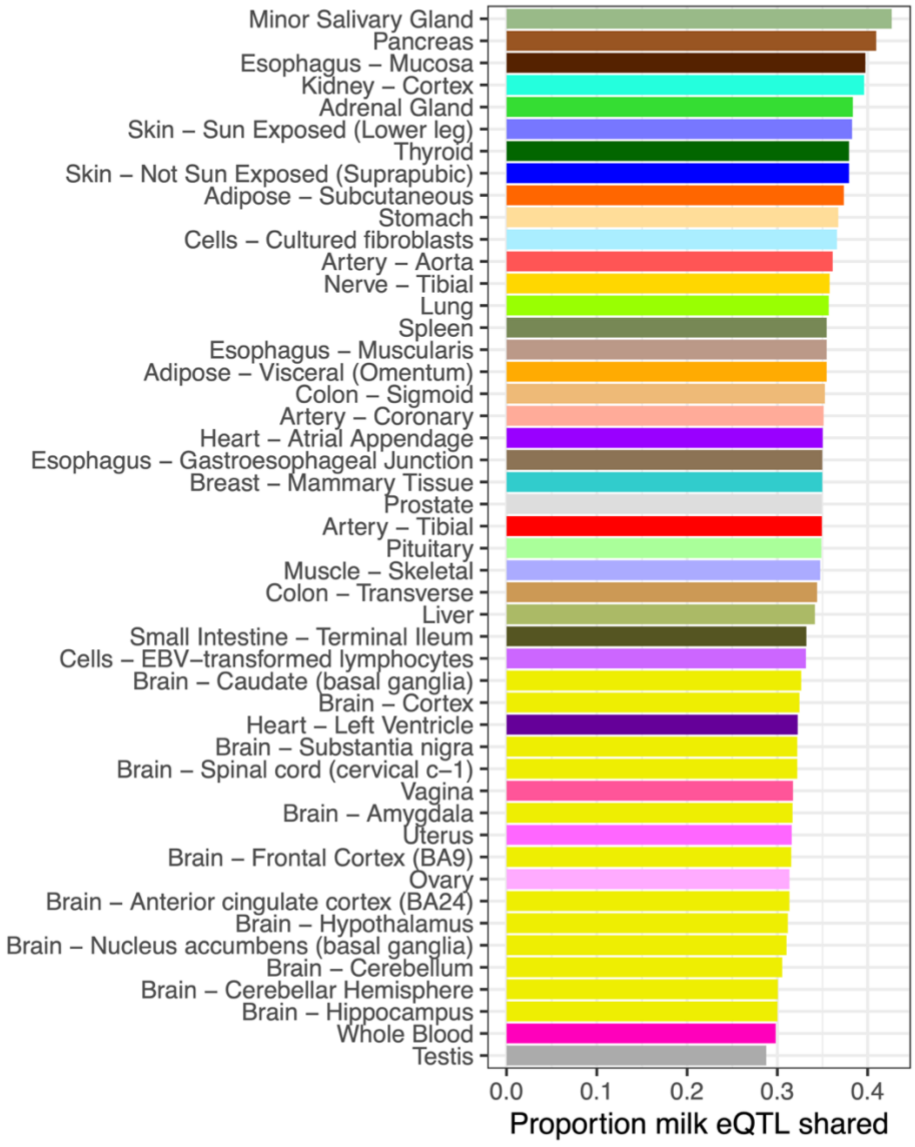

Figure S14.

Colocalization of a milk eQTL for *ATG10* and breast cancer GWAS locus, Related to Figure 2. *Top*: LocusZoom plots for milk eQTL and breast cancer GWAS association statistics. Each point represents a genetic variant, with genomic position along the x-axis and association P-values along the y-axis. Points are colored by their r^2 statistic with the lead variant denoted by a purple diamond. LD (r^2) was calculated using the European reference panel, at locuszoom.org. ***Bottom***: Posterior inclusion probabilities (PIP) from SuSiE fine-mapping of milk eQTL (blue) or breast cancer GWAS (green) statistics. Variants in the colocalized credible set for each trait are colored in gold. Each dot is a genetic variant, with genomic position along the x-axis and posterior inclusion probability (PIP) along the y-axis. The dashed vertical lines represent the genomic position of the lead variant(s) for each tissue's credible set(s).

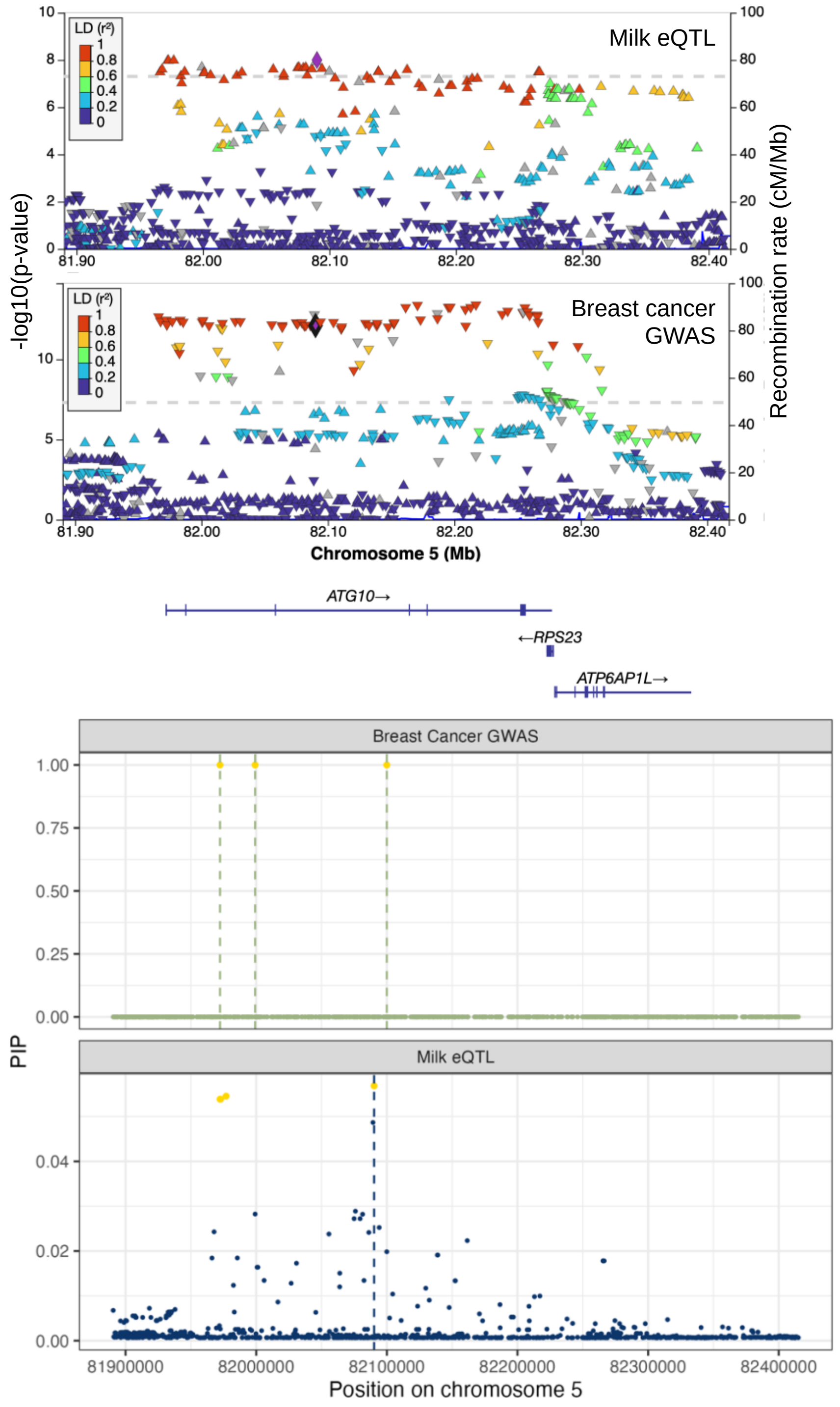

Figure S15.

Colocalization of a milk eQTL for *LMX1B* and breast cancer GWAS locus, Related to Figure 2. *Top*: LocusZoom plots for milk eQTL and breast cancer GWAS association statistics. Each point represents a genetic variant, with genomic position along the x-axis and association P-values along the y-axis. Points are colored by their r^2 statistic with the lead variant denoted by a purple diamond. LD (r^2) was calculated using the European reference panel, at locuszoom.org. ***Bottom***: Posterior inclusion probabilities (PIP) from SuSiE fine-mapping of milk eQTL (blue) or breast cancer GWAS (green) statistics. Variants in the colocalized credible set for each trait are colored in gold. Each dot is a genetic variant, with genomic position along the x-axis and posterior inclusion probability (PIP) along the y-axis. The dashed vertical lines represent the genomic position of the lead variant(s) for each tissue's credible set(s).

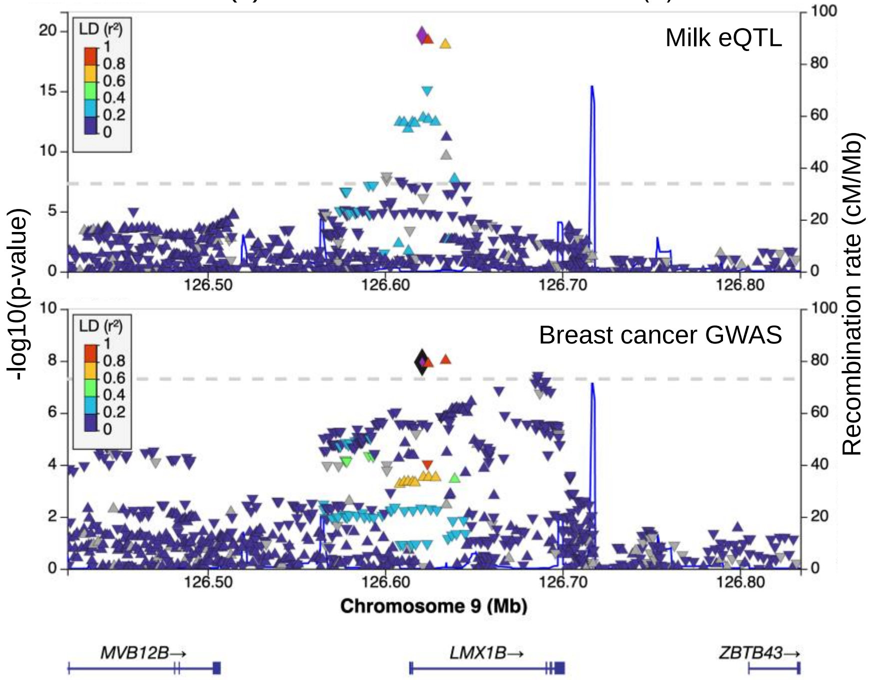

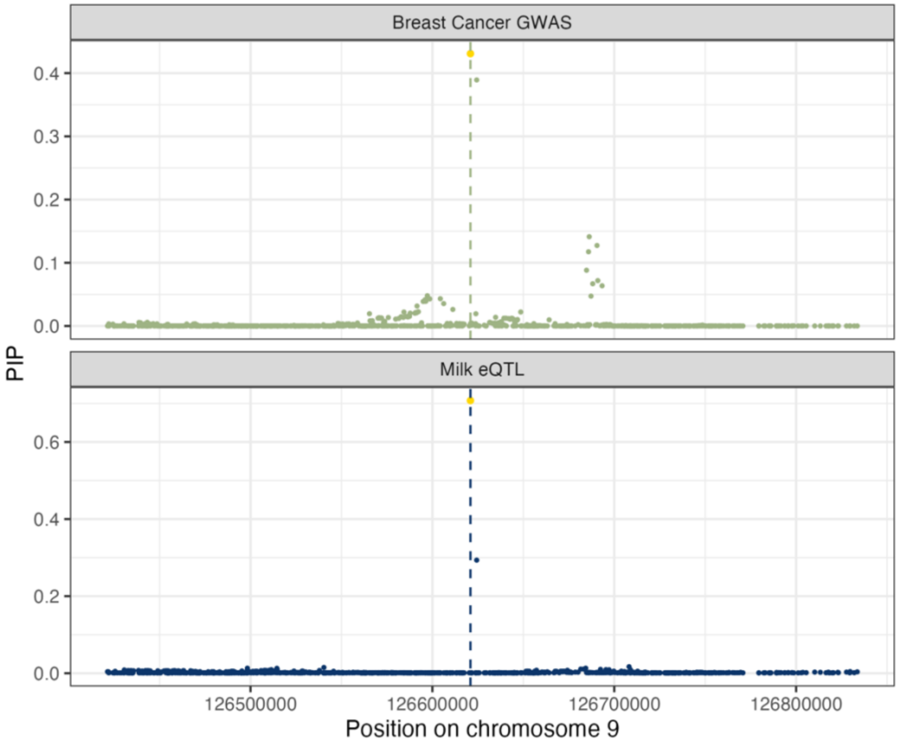

Figure S16.

Colocalization of a milk eQTL for *ARHGEF34P* and breast cancer GWAS locus, Related to Figure 2. ***Top***: LocusZoom plots for milk eQTL and breast cancer GWAS association statistics. Each point represents a genetic variant, with genomic position along the x-axis and association P-values along the y-axis. Points are colored by their r^2 statistic with the lead variant denoted by a purple diamond. LD (r^2) was calculated using the European reference panel, at locuszoom.org. ***Bottom***: Posterior inclusion probabilities (PIP) from SuSiE fine-mapping of milk eQTL (blue) or breast cancer GWAS (green) statistics. Variants in the colocalized credible set for each trait are colored in gold. Each dot is a genetic variant, with genomic position along the x-axis and posterior inclusion probability (PIP) along the y-axis. The dashed vertical lines represent the genomic position of the lead variant(s) for each tissue's credible set(s).

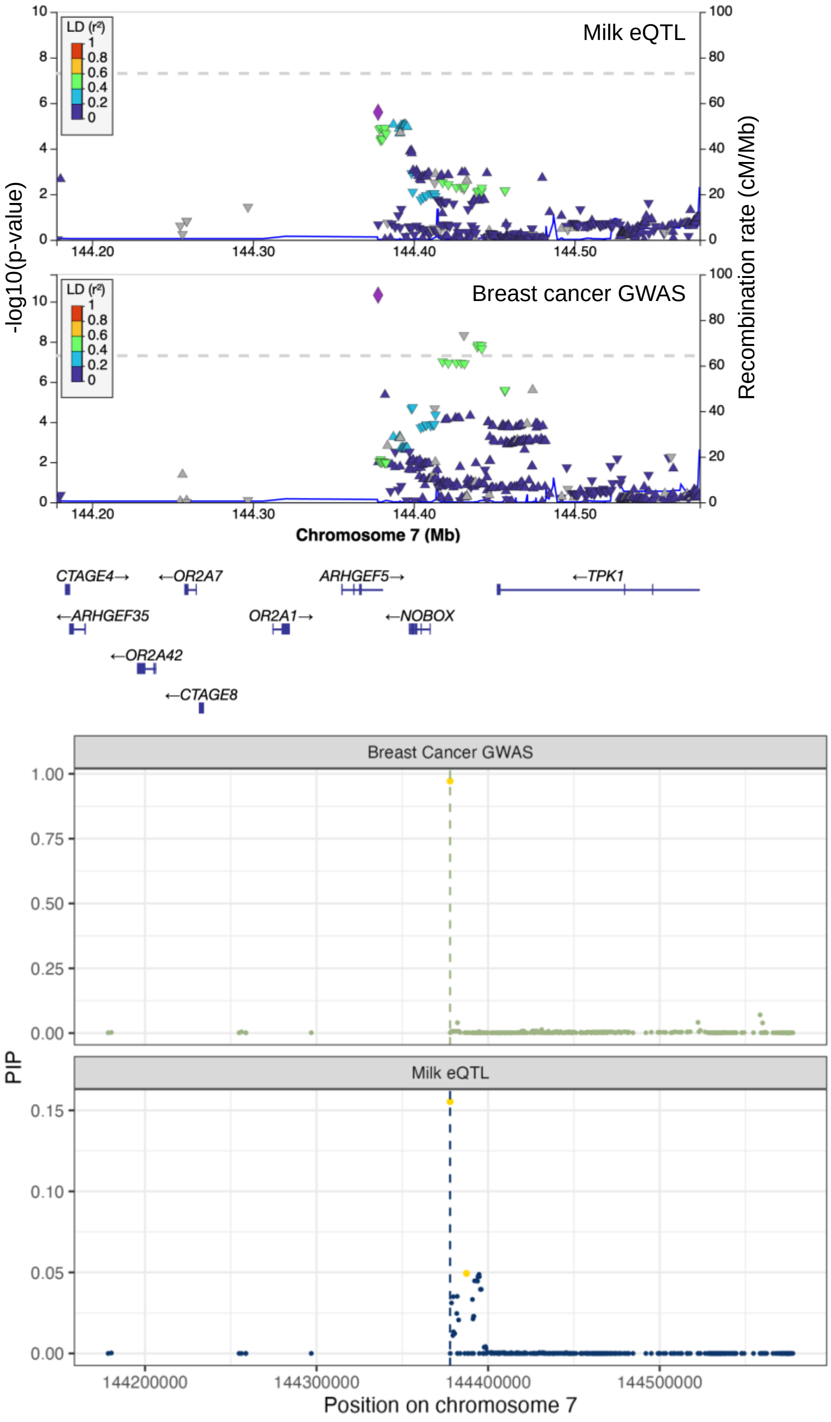

Figure S17.

Colocalization of a milk eQTL for *CDYL2* and breast cancer GWAS locus, Related to Figure 2. *Top*: LocusZoom plots for milk eQTL and breast cancer GWAS association statistics. Each point represents a genetic variant, with genomic position along the x-axis and association P-values along the y-axis. Points are colored by their r^2 statistic with the lead variant denoted by a purple diamond. LD (r^2) was calculated using the European reference panel, at locuszoom.org. ***Bottom***: Posterior inclusion probabilities (PIP) from SuSiE fine-mapping of milk eQTL (blue) or breast cancer GWAS (green) statistics. Variants in the colocalized credible set for each trait are colored in gold. Each dot is a genetic variant, with genomic position along the x-axis and posterior inclusion probability (PIP) along the y-axis. The dashed vertical lines represent the genomic position of the lead variant(s) for each tissue's credible set(s).

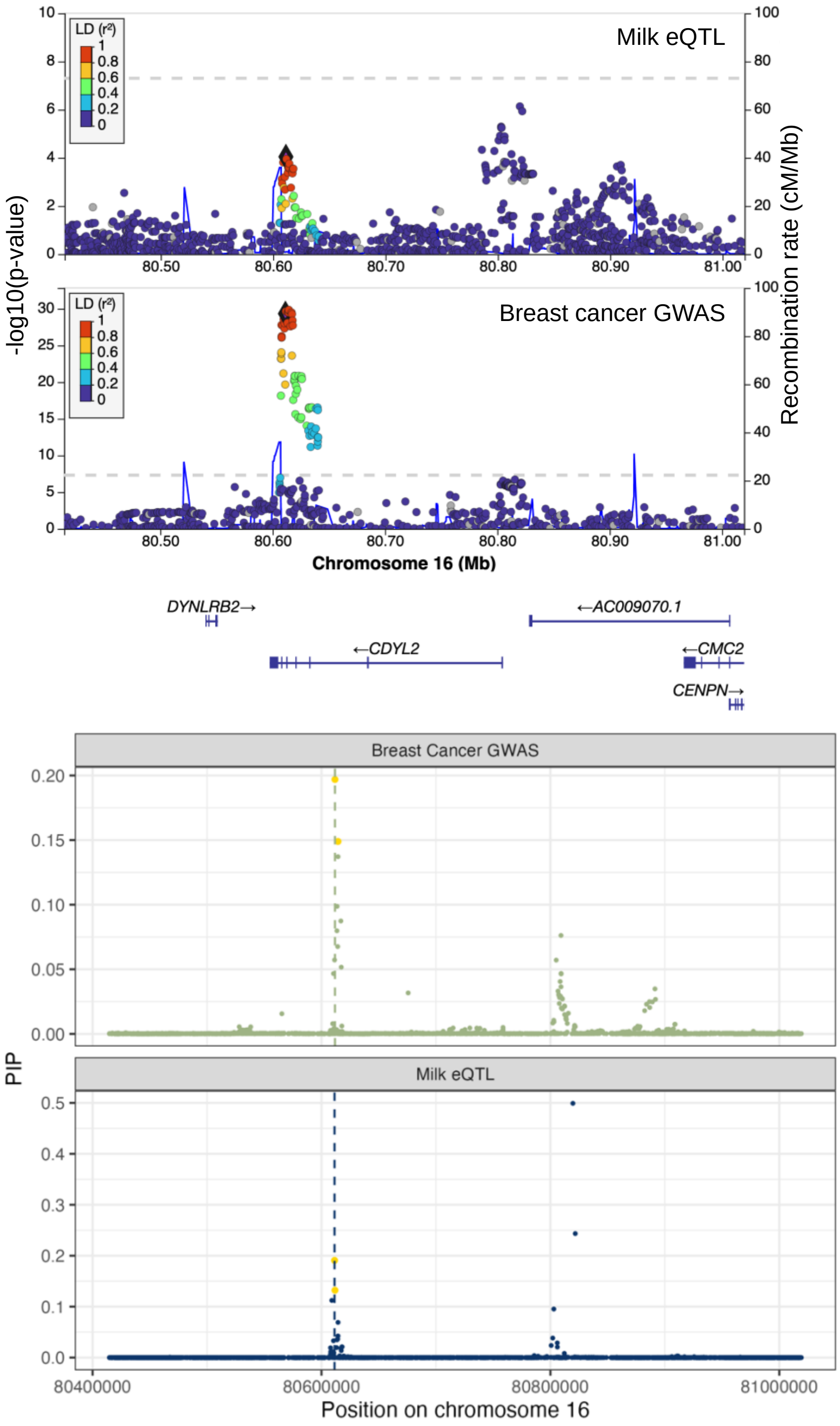

Figure S18.

Colocalization of a milk eQTL for *GTF2IP1* and breast cancer GWAS locus, Related to Figure 2. *Top*: LocusZoom plots for milk eQTL and breast cancer GWAS association statistics. Each point represents a genetic variant, with genomic position along the x-axis and association P-values along the y-axis. Points are colored by their r^2 statistic with the lead variant denoted by a purple diamond. LD (r^2) was calculated using the European reference panel, at locuszoom.org. ***Bottom***: Posterior inclusion probabilities (PIP) from SuSiE fine-mapping of milk eQTL (blue) or breast cancer GWAS (green) statistics. Variants in the colocalized credible set for each trait are colored in gold. Each dot is a genetic variant, with genomic position along the x-axis and posterior inclusion probability (PIP) along the y-axis. The dashed vertical lines represent the genomic position of the lead variant(s) for each tissue's credible set(s).

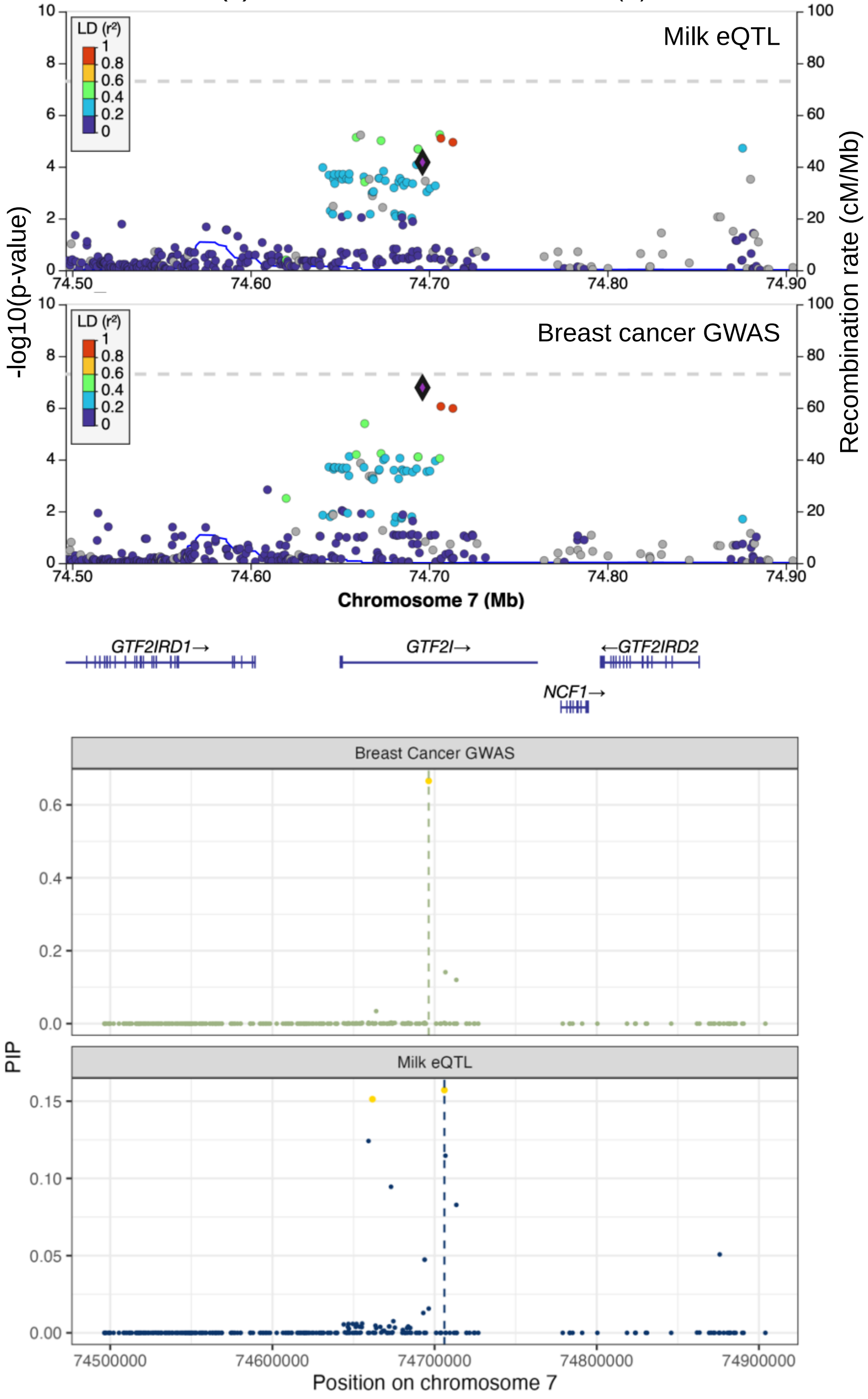

Figure S19.

Colocalization of a milk eQTL for *INHBB* and breast cancer GWAS locus, Related to Figure 2. ***Top***: LocusZoom plots for milk eQTL and breast cancer GWAS association statistics. Each point represents a genetic variant, with genomic position along the x-axis and association P-values along the y-axis. Points are colored by their r^2 statistic with the lead variant denoted by a purple diamond. LD (r^2) was calculated using the European reference panel, at locuszoom.org. ***Bottom***: Posterior inclusion probabilities (PIP) from SuSiE fine-mapping of milk eQTL (blue) or breast cancer GWAS (green) statistics. Variants in the colocalized credible set for each trait are colored in gold. Each dot is a genetic variant, with genomic position along the x-axis and posterior inclusion probability (PIP) along the y-axis. The dashed vertical lines represent the genomic position of the lead variant(s) for each tissue's credible set(s).

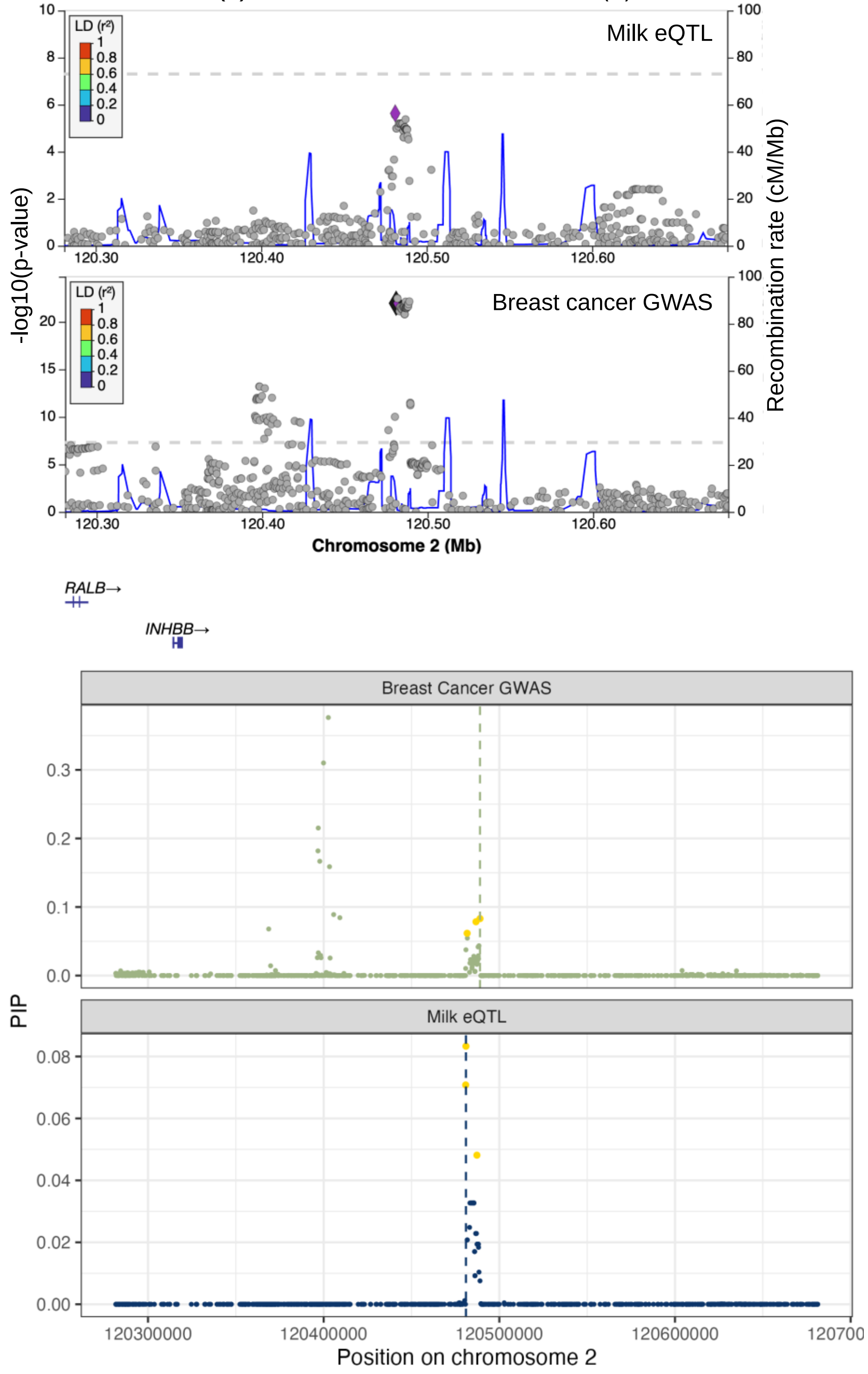

Figure S20.

Colocalization of *LMX1B* eQTLs in milk and tibial nerve tissue, Related to Figure 2. A) Locus plot of eQTL association statistics for milk (green) and tibial nerve (blue, from GTEx). Each point is a genetic variant, with genomic position on the x-axis and -log10(P-value) on the y-axis. Vertical dashed lines represent the lead variants for eQTL credible sets (milk has one credible set, tibial nerve has two). **B)** Posterior inclusion probabilities (PIP) from SuSiE[35] fine-mapping of milk eQTL (green) or tibial nerve eQTL (blue) statistics. The dashed vertical line represents the genomic position of the lead variant for each credible set. Variants in the colocalized credible set for each trait are colored in gold. For the milk credible set and secondary tibial nerve credible set, PP.H4 = 0.60 and PP.H3 = 0.13, passing our threshold of PP.H3/(PP.H4+PP.H3) > 0.8.

A)

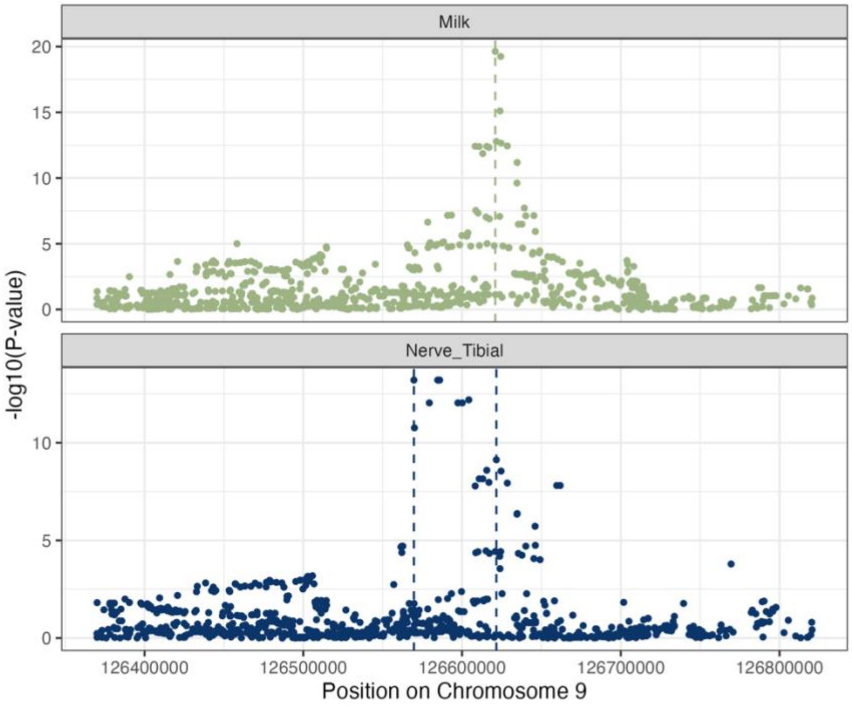

B)

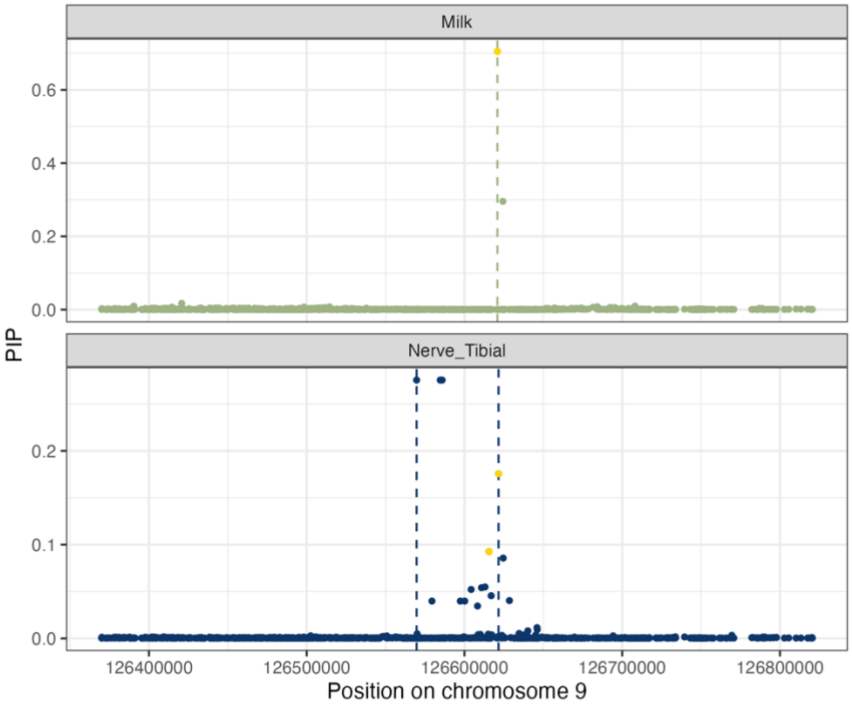

Figure S21.

HMO concentrations in secretors and non-secretors, Related to Figure 3. Distributions of HMO concentrations, grouped by secretor (blue) and non-secretor (red) individuals.

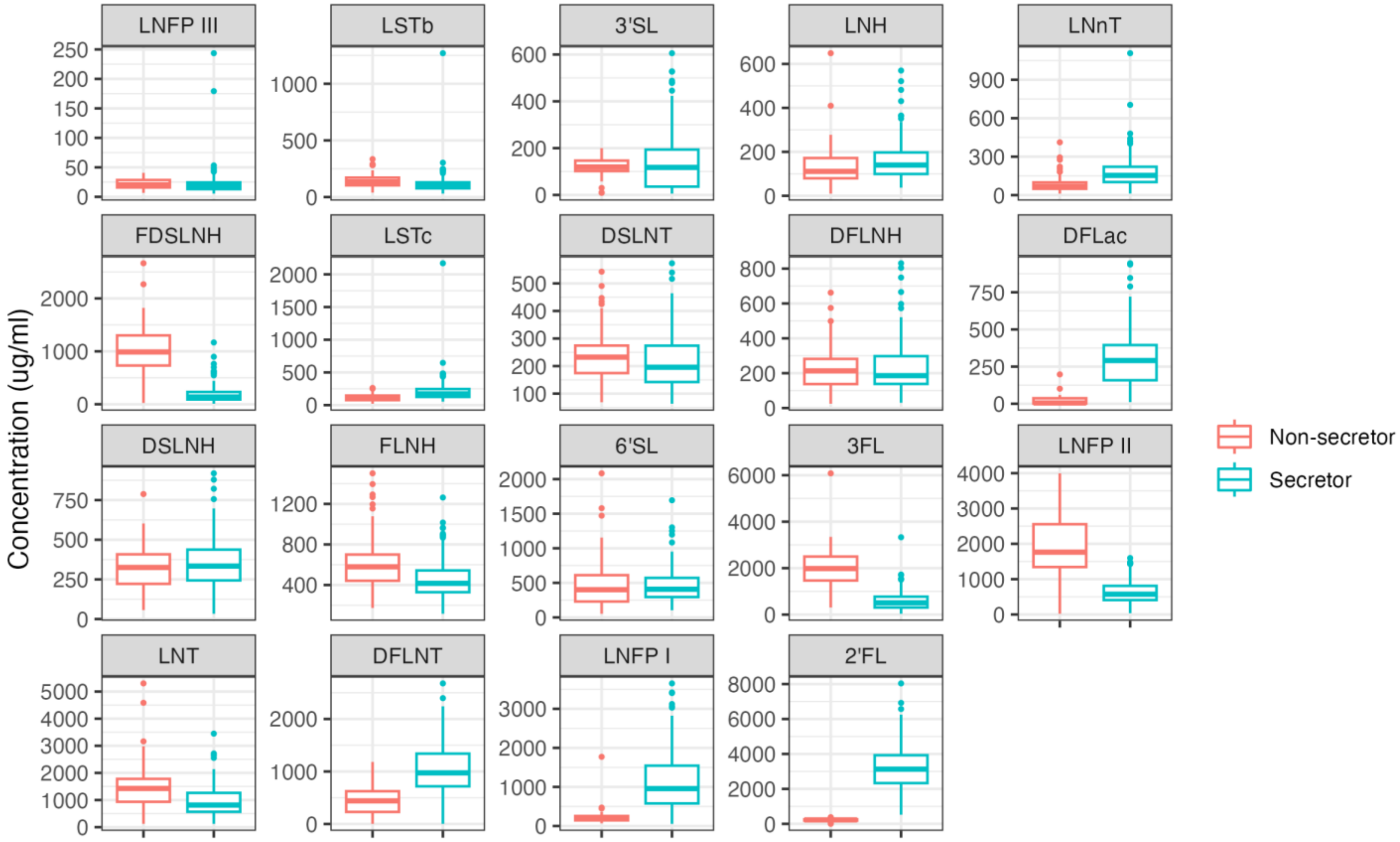

Figure S22.

Associations between *FUT2* gene expression in milk and HMO concentrations, related to Figure 3. Associations between normalized FUT2 expression (x-axis) and the normalized concentration (y-axis) of three HMOs (2'FL: Beta = 0.12, P = 0.01; LNFP-II: Beta = -0.12, P = 0.03; LNH: Beta = 0.14, P = 0.04). Regression statistics are for secretor individuals only. Secretors are shown in orange and non-secretors in light green.

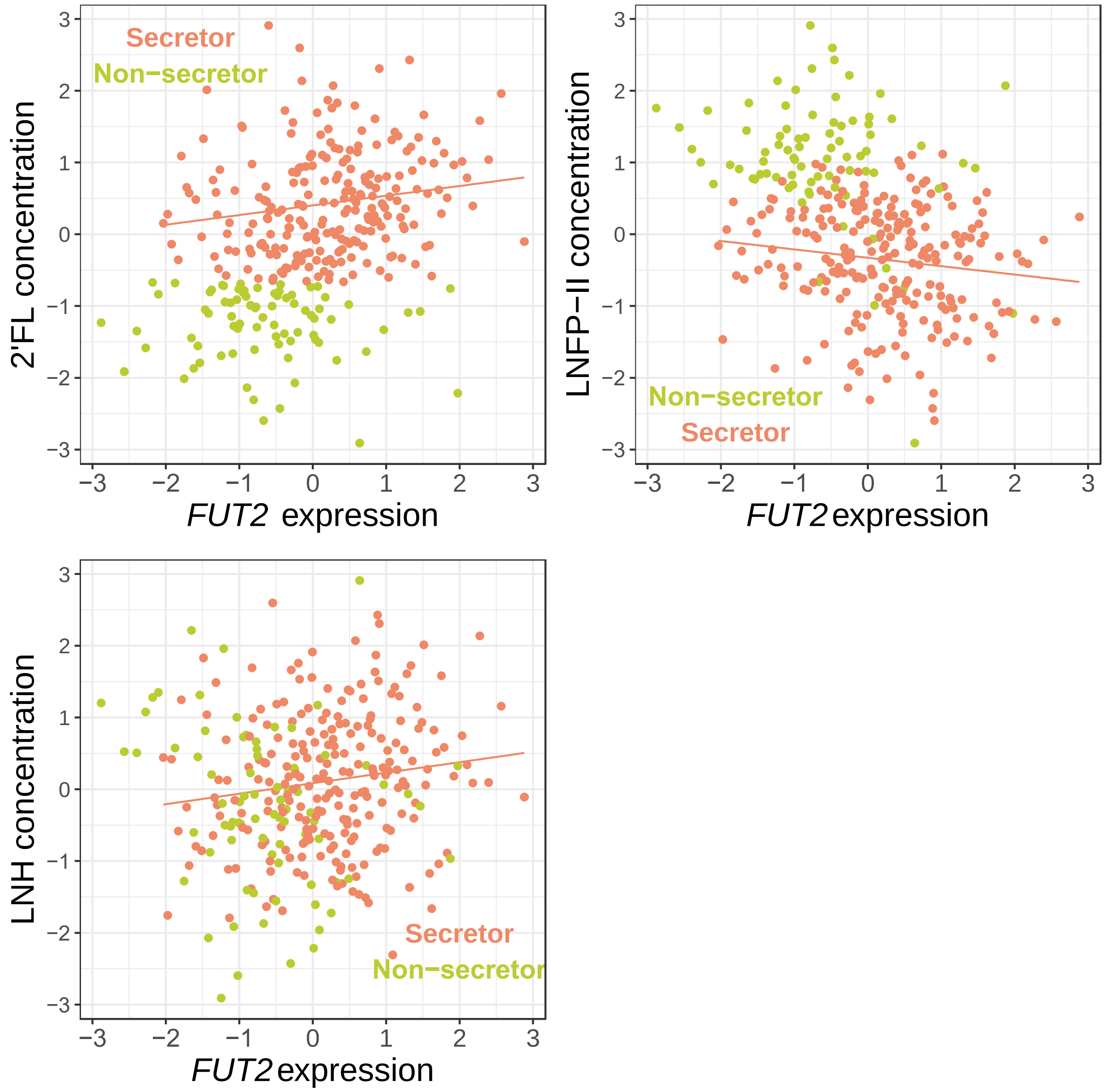

Figure S23.

Association between *GCNT3* gene expression in milk and FLNH concentration, related to Figure 3. Correlation between normalized GCTN3 expression and normalized FLNH concentration. Secretors are shown in orange and non-secretors in light green. To visualize the positive correlation in both secretors and non-secretors, regression lines are shown for secretors and non-secretors separately, but the relationship was assessed using all individuals with secretor status as a covariate in edgeR as described in Materials and Methods. Log fold-change= 0.33, P=3.3x10-7, q-value=4.5x10-4.

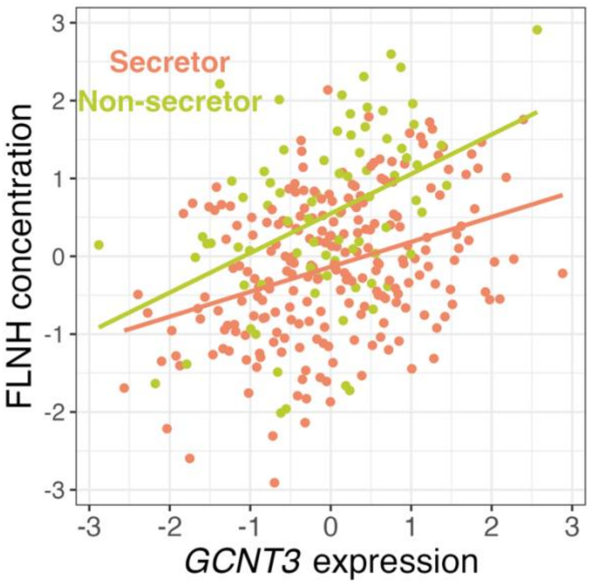

Figure S24.

Overview of infant fecal metagenomic data, Related to Figure 4. Infant metagenomic data summarized at the taxonomic level. **(A)** Bar plots showing the relative abundances of bacterial genera, grouped by 1-month (light blue, N=169) and 6-month (dark blue, N=155) samples. Each bar represents a sample. **(B)** Values of PC2 (y-axis; principal component 2 in Fig 4A) grouped by sample time point (x-axis). There was a significant difference between the two timepoints, using a linear mixed effects model with sample time point and delivery mode as fixed effects and subject ID as a random effect (timepoint effect est. = 2.5, $P = 1.5 \times 10^{-19}$). **(C)** Scatter plot of PC1 vs. PC2, as in Fig. 4A, but colored by delivery mode: vaginal (light purple) or cesarean (dark purple). Both 1-month and 6-month samples are plotted. **(D)** Values of PC1 (y-axis; principal component 1 in Figs. 4A and S6C) grouped by delivery mode (x-axis). There was a significant difference in PC1 score between the two delivery mode groups, using a linear mixed effects model with sample time point and delivery mode as fixed effects and subject ID as a random effect (cesarean effect est. = -1.8, $P = 4.8 \times 10^{-3}$).

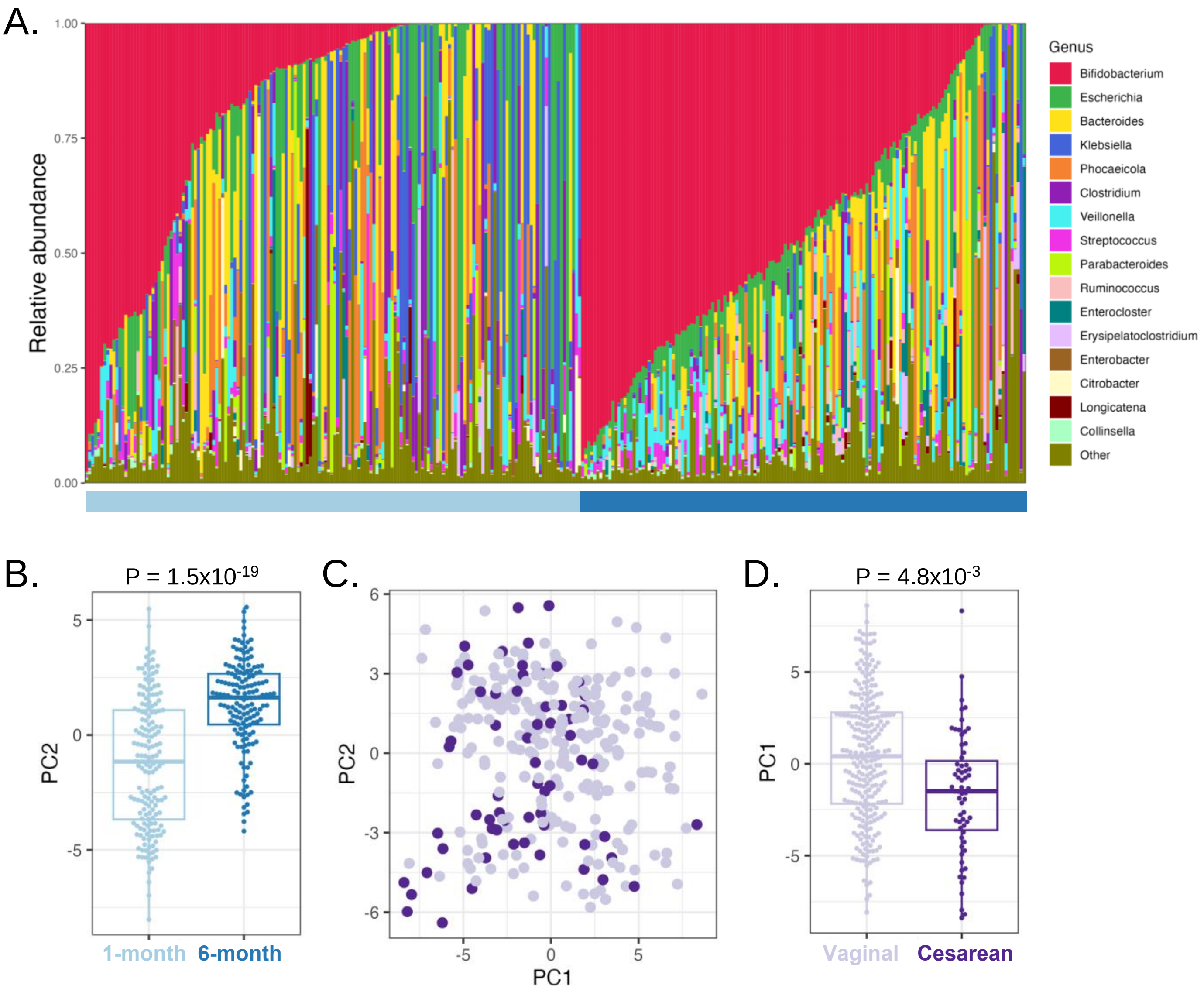

Table S2.

Overview of MILK study traits included in differential gene expression analyses, Related to Figure 1.

Trait: trait; **N**: sample size of trait for normalization (for DEG analysis, only samples with all trait info were used, N=171 for lactose/fat, N=269 for all other traits); **Mean**: sample mean, **Median**: sample median, **Min**: sample minimum; **Max**: sample maximum; **pct2.5**: sample 2.5 percentile; **pct97.5**: sample 97.5 percentile; **Units**: units of measurement.

Trait	N	Mean	Median	Min	Max	pct2.5	pct97.5	Units
Milk CRP	279	214.78	101.80	4.30	1877.04	12.65	1244.15	ng/ml
Milk glucose	279	30.13	29.71	1.88	67.13	11.39	53.84	mg/dl
Milk IL-6	276	19.05	3.87	0.12	1084.20	0.54	129.86	pg/ml
Milk insulin	279	29.89	25.30	1.61	119.30	6.96	80.73	IU/ml
Milk leptin	279	713.02	533.26	48.14	4864.40	121.88	2278.34	pg/ml
Milk volume	314	69.17	61.00	5.00	225.00	12.83	164.00	ml
Milk fat	173	4.78	4.68	1.23	8.19	2.51	7.09	%
Gestational diabetes	315	0.10	0.00	0.00	1.00	0.00	1.00	status
Gestational weight gain	280	12.29	12.25	-6.81	38.10	0.89	24.50	kg
Milk lactose	173	6.60	6.62	5.99	7.07	6.22	7.01	%
Maternal pre-pregnancy BMI	314	27.05	26.10	18.50	56.75	19.53	40.12	kg/m^2
Maternal age	314	31.38	31.00	21.00	42.00	23.00	40.00	years
Parity	284	1.18	1.00	0.00	6.00	0.00	3.93	# previous births

Table S4.

Correlation between maternal/milk traits and gene expression in either the top or bottom half of samples by RIN, Related to STAR Methods.

Trait: trait tested; **nSig.allSamp**: number significant genes when all samples were included; **nSig.botRIN**: number significant genes when the bottom half of samples by RIN were included; **nSig.topRIN**: number of significant genes when the top half of samples by RIN were included; **botRINsig.corr**: Pearson correlation coefficient comparing logFC from top & bottom half of samples, with only genes significant in the bottom half sample; **botRINsig.corrP**: P-value of botRINsig.corr estimate; **allgenes.corr**: Pearson correlation coefficient comparing logFC from top & bottom half of samples, including all genes; **allgenes.corrP**: P-value of allgenes.corr estimate.

Trait	nSig.allSamp	nSig.botRIN	nSig.topRIN	botRINsig.corr	botRINsig.corrP	allgenes.corr	allgenes.corrP
milk glucose	1194	79	606	0.86	2.97E-24	0.33	4.91E-300
milk IL-6	980	285	492	0.91	6.74E-109	0.68	0.00E+00
milk insulin	785	5	689	0.97	7.22E-03	0.23	2.65E-144
milk lactose	89	38	0	0.45	5.05E-03	0.30	8.91E-247
parity	88	11	27	0.82	1.81E-03	0.14	1.37E-54
milk volume	78	13	60	0.82	6.17E-04	0.13	4.07E-44
maternal age	16	7	8	0.39	3.89E-01	0.00	7.12E-01
milk leptin	12	3	4	NA	NA	-0.01	4.09E-01
maternal pre-pregnancy BMI	10	0	1	NA	NA	0.12	5.80E-36
milk fat	9	0	1	NA	NA	0.10	1.62E-27
milk CRP	7	2	1	NA	NA	-0.03	2.55E-03
gestational weight gain	6	4	5	NA	NA	0.02	4.09E-02

Table S14.

HMO abbreviations and full names, Related to Figure 3.

**Abbreviation**	**Full name**
LSTc	sialyl-LNT c
LSTb	sialyl-LNT b
LNT	lacto-N-tetraose
LNnT	lacto-N-neotetraose
LNH	lacto-N-hexaose
LNFP-III	lacto-N-fucopentaose III
LNFP-II	lacto-N-fucopentaose II
LNFP-I	lacto-N-fucopentaose I
FLNH	fucosyllacto-N-hexaose
FDSLNH	fucodisialyllacto-lacto-N-hexaose
DSLNT	disialyllacto-N-tetraose
DSLNH	disialyllacto-N-hexaose
DFLNT	difucosyllacto-LNT
DFLNH	difucosyllacto-N-hexaose
DFLac	difucosyllactose
6'SL	6′-sialyllactose
3'SL	3′-sialyllactose
2'SL	2′-sialyllactose
2'FL	2′-fucosyllactose
